# Ultrasmall iron oxide nanoparticles cause significant toxicity by specifically inducing acute oxidative stress to multiple organs

**DOI:** 10.1186/s12989-022-00465-y

**Published:** 2022-03-29

**Authors:** Lin Wu, Wen Wen, Xiaofeng Wang, Danhua Huang, Jin Cao, Xueyong Qi, Song Shen

**Affiliations:** 1 Affiliated Hospital of Jiangsu University, Zhenjiang, 212001 China; 2 School of Pharmaceutical Science, Jiangsu University, 308 Xuefu Road, Zhenjiang, 212013 China

**Keywords:** Iron oxide, ROS, Ultrasmall nanoparticles, Toxicity, Oxidative stress

## Abstract

**Background:**

Iron oxide nanoparticles have been approved by food and drug administration for clinical application as magnetic resonance imaging (MRI) and are considered to be a biocompatible material. Large iron oxide nanoparticles are usually used as transversal (*T*_2_) contrast agents to exhibit dark contrast in MRI. In contrast, ultrasmall iron oxide nanoparticles (USPIONs) (several nanometers) showed remarkable advantage in longitudinal (*T*_1_)-weighted MRI due to the brighten effect. The study of the toxicity mainly focuses on particles with size of tens to hundreds of nanometers, while little is known about the toxicity of USPIONs.

**Results:**

We fabricated Fe_3_O_4_ nanoparticles with diameters of 2.3, 4.2, and 9.3 nm and evaluated their toxicity in mice by intravenous injection. The results indicate that ultrasmall iron oxide nanoparticles with small size (2.3 and 4.2 nm) were highly toxic and were lethal at a dosage of 100 mg/kg. In contrast, no obvious toxicity was observed for iron oxide nanoparticles with size of 9.3 nm. The toxicity of small nanoparticles (2.3 and 4.2 nm) could be reduced when the total dose was split into 4 doses with each interval for 5 min. To study the toxicology, we synthesized different-sized SiO_2_ and gold nanoparticles. No significant toxicity was observed for ultrasmall SiO_2_ and gold nanoparticles in the mice. Hence, the toxicity of the ultrasmall Fe_3_O_4_ nanoparticles should be attributed to both the iron element and size. In the in vitro experiments, all the ultrasmall nanoparticles (< 5 nm) of Fe_3_O_4_, SiO_2_, and gold induced the generation of the reactive oxygen species (ROS) efficiently, while no obvious ROS was observed in larger nanoparticles groups. However, the ·OH was only detected in Fe_3_O_4_ group instead of SiO_2_ and gold groups. After intravenous injection, significantly elevated ·OH level was observed in heart, serum, and multiple organs. Among these organs, heart showed highest ·OH level due to the high distribution of ultrasmall Fe_3_O_4_ nanoparticles, leading to the acute cardiac failure and death.

**Conclusion:**

Ultrasmall Fe_3_O_4_ nanoparticles (2.3 and 4.2 nm) showed high toxicity in vivo due to the distinctive capability in inducing the generation of ·OH in multiple organs, especially in heart. The toxicity was related to both the iron element and size. These findings provide novel insight into the toxicology of ultrasmall Fe_3_O_4_ nanoparticles, and also highlight the need of comprehensive evaluation for their clinic application.

**Graphical Abstract:**

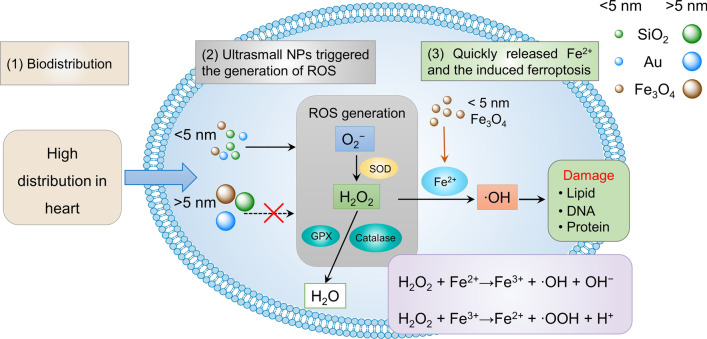

**Supplementary Information:**

The online version contains supplementary material available at 10.1186/s12989-022-00465-y.

## Introduction

The unique physical and chemical features make the superparamagnetic iron oxide nanoparticles (SPIONs) to be a promising platform for targeting drug delivery [1–3], magnetic resonance imaging (MRI) [4], magnetic separation [5], photothermal therapy [6, 7], and magnetic hyperthermia [8, 9]. Recently, the SPIONs have also been found to be effective in treatment of leukaemia [10] and in the enhancement of chemotherapeutic activity [11]. For the clinical application in MRI, various commercial SPIONs contrast agents, including Feridex (Ferumoxides, 120–180 nm), Resovist (Ferucarbotran, 45–60 nm), and Feraheme (ferumoxytol, 20–30 nm), have been approved by food and drug administration (FDA) [12–14]. However, the SPIONs used in these studies are generally larger than 20 nm, which tend to be retained by the reticuloendothelial system (RES), resulting in shorter blood half-life [15]. Meanwhile, the large-sized SPIONs exhibit high transverse relaxivity (*r*2) and are usually functioned as negative *T*_2_ contrast agents to show darkened areas, which lower the image differentiation [16].

Ultrasmall iron oxide nanoparticles (USIONPs) exhibit high longitudinal relaxivity (*r*_1_), presenting a good candidate for positive *T*_1_-weighted imaging [17]. In addition, the NPs could be efficiently cleared from kidney after *i.v.* administration once the size was controlled below 5.5 nm [18]. Although USPIONs have attracted great attention, the bio-safety is not completely understood and it is quite necessary to study the toxicity, since various kinds of nanoparticles demonstrate size-dependent toxicity in vitro and vivo. Gold nanoparticles with size smaller than 5 nm are much more toxic to human embryonic stem cells [19] and mouse fibroblasts [20]. Silica also indicated size-dependent toxicity [21, 22] and size-dependent activity that only ultrasmall NPs (< 5 nm) could activate the mononuclear cells [23]. The toxicity might be related to the large surface, quick dissolution, and good penetration [24]. To date, the toxicity of USPIONs is debatable and needs to be further clarified.

We evaluated the toxicity of USPIONs with three sizes (2.3, 4.2, and 9.3 nm) in vitro and in vivo using gold and silica NPs as controls. The result was pretty impressive that small-sized USPIONs (2.3 and 4.2 nm) were highly toxic and showed lethal dose of 100 mg/kg, while the mice treated with gold NPs, silica NPs, or large-sized iron oxide NPs (9.3 nm) were alive. It is reasonable to deduce that the strong toxicity of USPIONs is related to both the ultrasmall size and the iron element. Here, we investigated the possible lethal factors associated with the size and iron element, including the biodistribution of the NPs, USPIONs induced generation of ROS, and ferroptosis based on Fe^2+^ and radicals (·OH).

## Results and discussion

### Construction and characterization of the Fe_3_O_4_, SiO_2_, and Au NPs

**Fig. 1:**
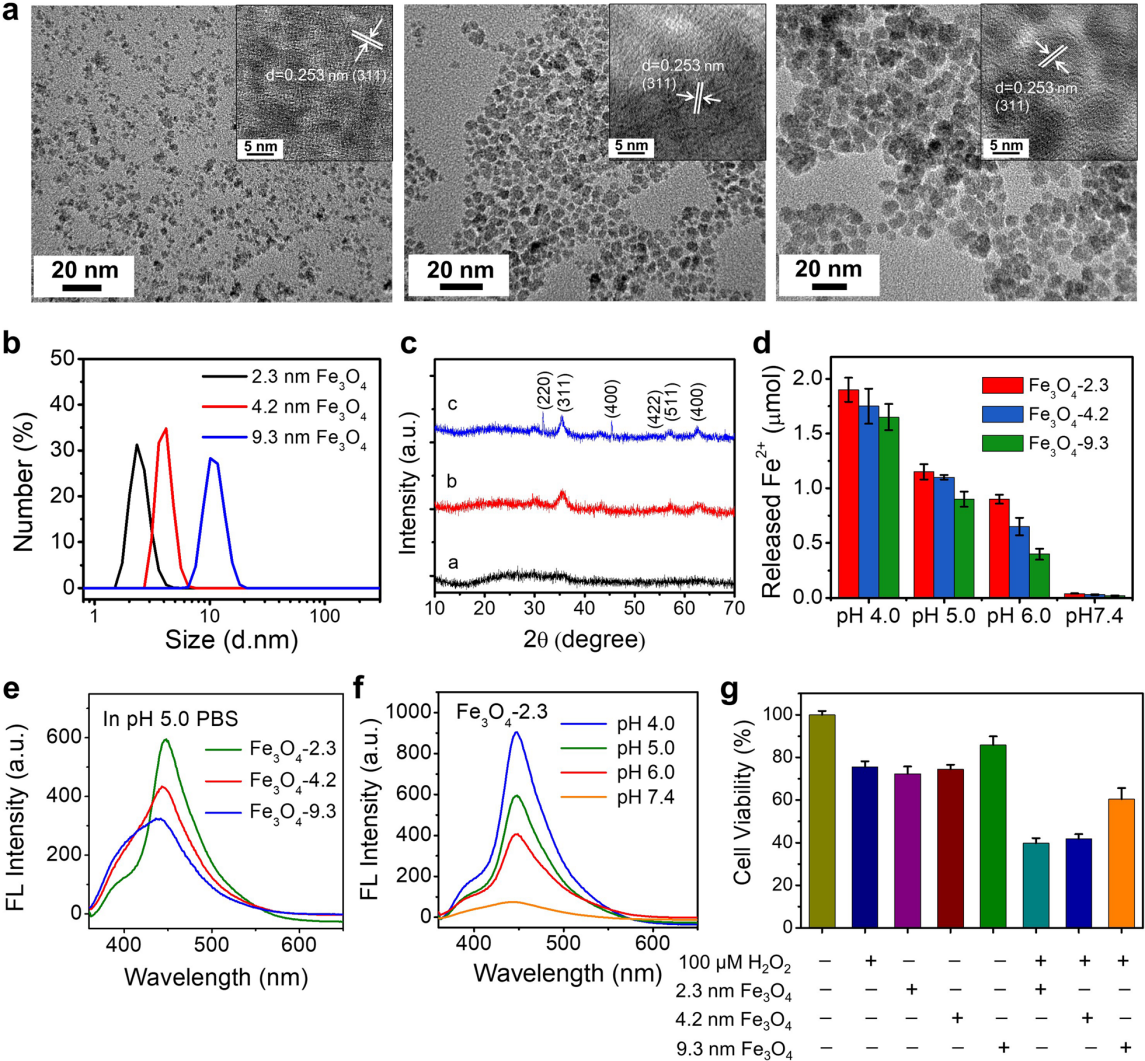
Characterizations of Fe_3_O_4_ NPs. **a** TEM images of the Fe_3_O_4_ NPs (2.3 nm (left), 4.2 nm (middle) and 9.3 nm (right)). The inserts are the high magnification images. **b** Particle size distribution of Fe_3_O_4_ NPs. **c** XRD patterns of Fe_3_O_4_ NPs (2.3 nm (bottom), 4.2 nm (middle) and 9.3 nm (up)). **d** The release of Fe^2+^ from Fe_3_O_4_ NPs (2.3 nm, 4.2 nm, and 9.3 nm) in PBS with different pH values (pH 4.0, 5.0, 6.0, and 7.4). **e** Detection of generated ·OH from different-sized Fe_3_O_4_ NPs in pH 5.0 PBS using 3-CCA as fluorescence indicator. **f** The generation of ·OH from 2.3 nm Fe_3_O_4_ NPs in PBS with different pH values (pH 4.0, 5.0, 6.0, and 7.4). **g** The cytotoxicity of Fe_3_O_4_ NPs (1000 μg/mL) to MCF-7 cells in the presence and absence of H_2_O_2_

Different-sized Fe_3_O_4_ NPs were synthesized using a modified solvothermal reaction with diethylene glycol, sodium acetate, and trisodium citrate as solvent, alkali source, and stabilizer, respectively. The particle size could be controlled by adjusting the reaction temperature and time. TEM and DLS were used to characterize the morphology and size of synthesized Fe_3_O_4_ NPs. As shown in Fig. 1a, b, the monodispersed Fe_3_O_4_ nanoparticles were spherical in shape with mean diameters of 2.3 nm, 4.2 nm and 9.3 nm, respectively. SiO_2_ NPs with average sizes of 2.4 nm, 4.2 nm, 7.3 nm and Au NPs with average sizes of 4.1 nm, 7.2 nm, 11.0 nm were synthesized and used as controls (Additional file 1: Figs. S1, S2). The lattice spacing of an individual nanocrystal from HRTEM was measured to be 0.253 nm, which could be assigned to the (311) planes in the inverse spinel-structured Fe_3_O_4_. The power X-ray diffraction (XRD) patterns (Fig. 1c) indicate that the positions of all diffraction peaks match well with standard Fe_3_O_4_ powder diffraction data (JCPDS card no. 19-0629). These results suggest that the three kinds of Fe_3_O_4_ NPs have the same lattice structure.

To investigate the release of Fe^2+^ from Fe_3_O_4_ NPs, different-sized Fe_3_O_4_ NPs were dispersed in PBS with various pH values (4.0, 5.0, 6.0, and 7.4). The amount of released Fe^2+^ was determined by the titration of potassium permanganate solution. Accelerated release of Fe^2+^ was observed in the acidic medium due to the pH sensitive dissolution of the iron oxide (Fig. 1d). The release rate was also influenced by the size of the NPs that smaller Fe_3_O_4_ NPs resulted in quicker release because of the relatively higher specific surface (Fig. 1d).

We then studied the Fenton reaction of H_2_O_2_ catalyzed by Fe_3_O_4_ NPs. The generated ·OH group was determined using coumarin-3-carboxylic acid (3-CCA) as an indicator, which was non-fluorescent and could be hydroxylated by ·OH to generate fluorescent 7-hydroxycoumarin [25]. Different-sized Fe_3_O_4_ NPs were incubated with H_2_O_2_ at pH 5.0 for 2 h and then the fluorescence was determined. As shown in Fig. 1e, brightest fluorescence was observed in 2.3 nm Fe_3_O_4_ NPs group, indicating the superior catalytic activity for H_2_O_2_. Considering the pH-dependent release of Fe^2+^ from Fe_3_O_4_ NPs, it was reasonable to assume that the generation of ·OH was pH dependent. This assumption was validated by the results in Fig. 1f. There was negligible ·OH generated at pH 7.4, while strongest signal was detected in pH 4.0 medium. We then explored the effect of Fe_3_O_4_ NPs on the cytotoxicity of H_2_O_2_ to human breast carcinoma cell line (MCF-7) by MTT assay. As shown in Fig. 1g and Additional file 1: Fig. S3, H_2_O_2_ showed obvious toxicity to cells and the viability was decreased to 74.3% after exposing to H_2_O_2_ at a concentration of 100 μM. In the presence of 2.3 nm Fe_3_O_4_ NPs, the cell viability was further decreased to 42.1%. The enhanced toxicity should be attributed to the generation of ·OH. Similar toxicity was observed in 4.2 nm Fe_3_O_4_ NPs group. The synergetic toxicity of could be significantly inhibited by the antioxidant glutathione (GSH) (Additional file 1: Fig. S3). In absence of H_2_O_2_, we also observed the cytotoxicity of 2.3 nm and 4.2 nm Fe_3_O_4_ NPs to the MCF-7 cells, while no obvious toxicity was observed for 9.3 nm Fe_3_O_4_ (Additional file 1: Fig. S3). To explain this phenomenon, we proposed a hypothesis that the Fe_3_O_4_ NPs with size < 5 nm could induce the generation of ROS including O_2_^−^, and H_2_O_2_, which were subsequently catalyzed to highly toxic ·OH to destroy the cells.

### The uptake of the NPs in cells

**Fig. 2:**
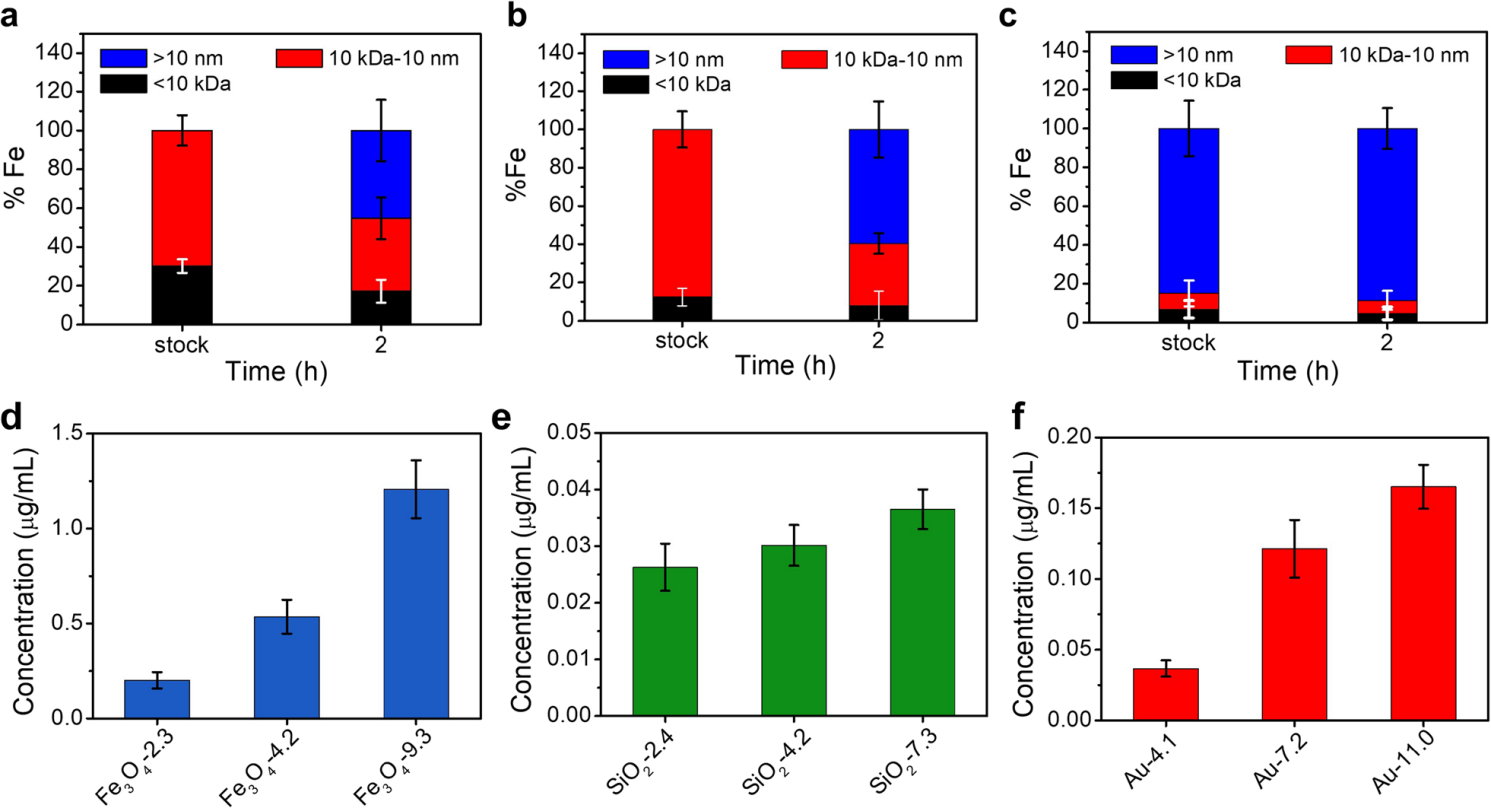
Percentage of iron that passed through 10 kDa and 10 nm ultrafilters of 2.3 nm (**a**), 4.2 nm (**b**), and 9.3 nm Fe_3_O_4_ NPs after incubation with DMEM media at a concentration of 100 μM Fe for 2 h. Cellular concentrations of Fe (**e**), Si (**f**), and Au (**g**) after incubation with different-sized Fe_3_O_4_, SiO_2_, and Au NPs at the same concentration of 50 μg/mL for 2 h

Most nanoparticles are unstable in ion containing media, readily forming aggregates due to the change of surface charge. We determined the size change of the NPs by ultrafiltration and elemental analysis. As shown in Fig. 2a, b, after incubation in the DMEM media for 2 h, the 2.3 nm and 4.2 nm Fe_3_O_4_ NPs showed significant aggregation that the amount of NPs with size > 10 nm was over 40%. The aggregation was also found in 9.3 nm Fe_3_O_4_ NPs (Fig. 2c). Although the aggregation happened, we could also observe the presence of NPs with size < 2.9 nm. Fe_3_O_4_, SiO_2_ and Au NPs showed size-dependent internalization in MCF-7 cells (Fig. 2d–f). After incubation at a fixed concentration of 50 μg/mL for 2 h, higher Fe/Si/Au conctents were observed in larger NPs treated cells. This phonomenon has been reported before [26].

The intracellular uptake of the different-sized NPs was characterized by LSCM. As presented in Additional file 1: Fig. S4, the 2.3 nm and 4.2 nm Fe_3_O_4_ NPs could enter the nuclei efficiently after incubation with cells for 0.5 h, while the 9.3 nm Fe_3_O_4_ NPs was observed outside the nuclei. Similar phenomenon was also found in SiO_2_ and Au NPs groups (Additional file 1: Figs. S5, S6). These results indicates only NPs with size < 5 nm can enter the nuclei since the nuclei pore is ~ 5 nm.

### The ultrasmall NPs induced generation of ROS in cells

**Fig. 3:**
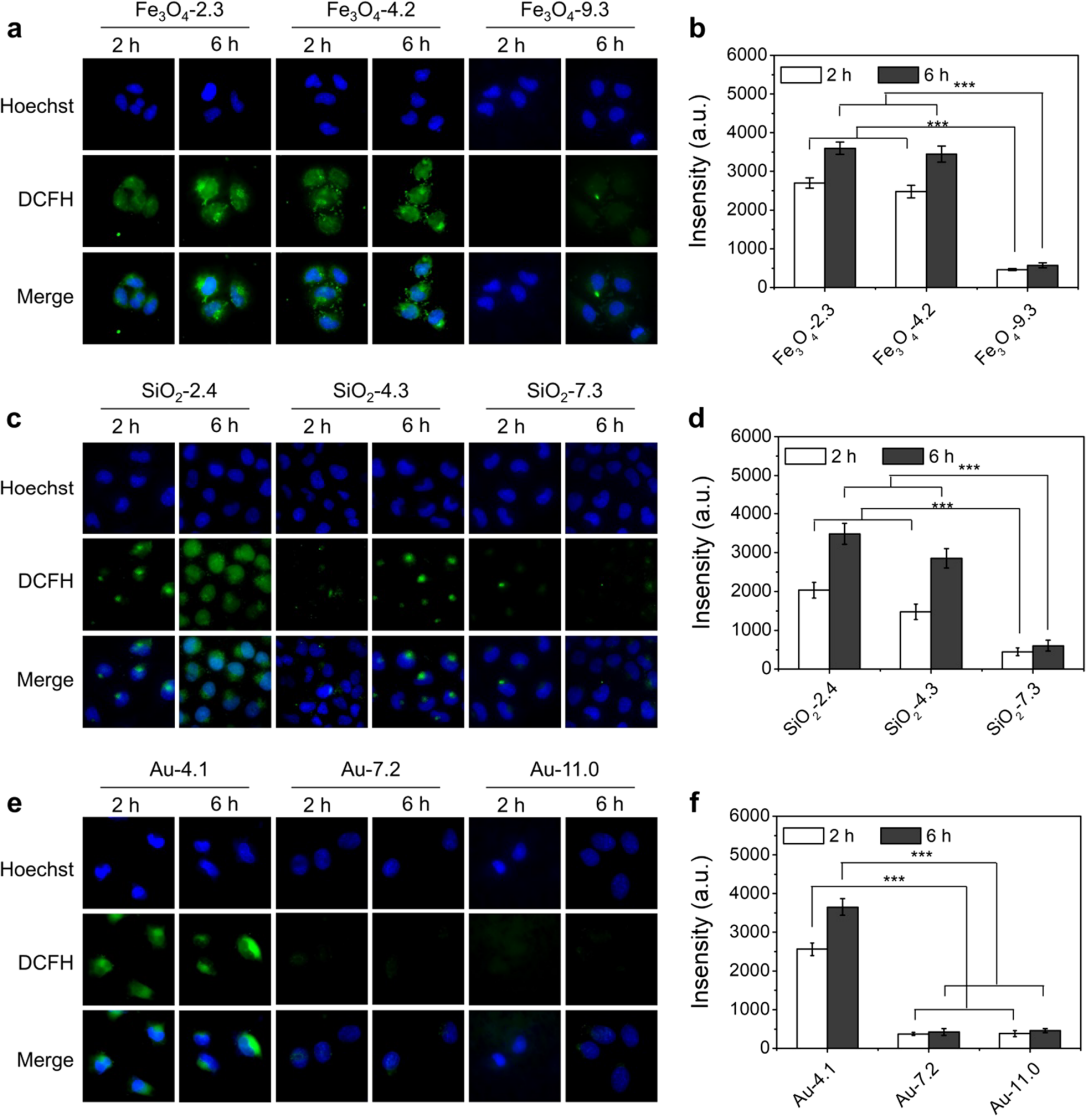
Detection of the generated ROS. Fluorescence images showing the ROS of MCF-7 cells after incubation with different-sized Fe_3_O_4_ (**a**), SiO_2_ (**c**), and Au (**e**) with a concentration of 500 μg/mL for 2 h and 6 h. Green fluorescence: DCFH-DA, an indicator of ROS; blue fluorescence: Hoechst 33,342. The corresponding fluorescence intensities of DCFH in MCF-7 cells after incubation with different-sized Fe_3_O_4_ (**b**), SiO_2_ (**d**), and Au (**f**) for 2 h and 6 h. All of the data were presented as the mean ± SD (n = 3). *P* values: ***P < 0.001

We investigated the NPs induced ROS in MCF-7 cells using DCFH-DA as a probe, which was non-fluorescent and could be oxidized by ROS to produce fluorescent DCF [27, 28]. In Fe_3_O_4_ groups, we could observe bright fluorescence in 2.3 nm and 4.2 nm Fe_3_O_4_ NPs treated MCF-7 cells (Fig. 3a, b). The strength of the fluorescence increased as the incubation time extended. By contrast, only extremely weak signal was detected in 9.3 nm Fe_3_O_4_ NPs group. Considering the relatively lower internalization of ultrasmall NPs, the elevated ROS should be attributed to the stimulation of the ultrasmall NPs. As for SiO_2_ group, size- and time-dependent fluorescence was also observed in the cells (Fig. 3c, d). ROS was hard to be detected in 7.3 nm SiO_2_ group. Similar phenomenon was observed in Au NPs treated cells that fluorescence was only observed in 4.1 nm NPs groups, while no obvious fluorescence was detected in 7.2 nm and 11 nm Au NPs treated cells (Fig. 3e, f). For the three different NPs, there was significant difference of the fluorescence intensity between the NPs of < 5 nm and > 5 nm groups, indicating the remarkable ROS induction ability of the ultrasmall NPs (< 5 nm). We also noticed that the DCF fluorescence in 2.3 nm and 4.2 nm Fe_3_O_4_ NPs was located within the whole cells including the nucleus. This is probably because only ultrasmall NPs could enter the nucleus efficiently.

### Identification of the generated ROS in cells

**Fig. 4:**
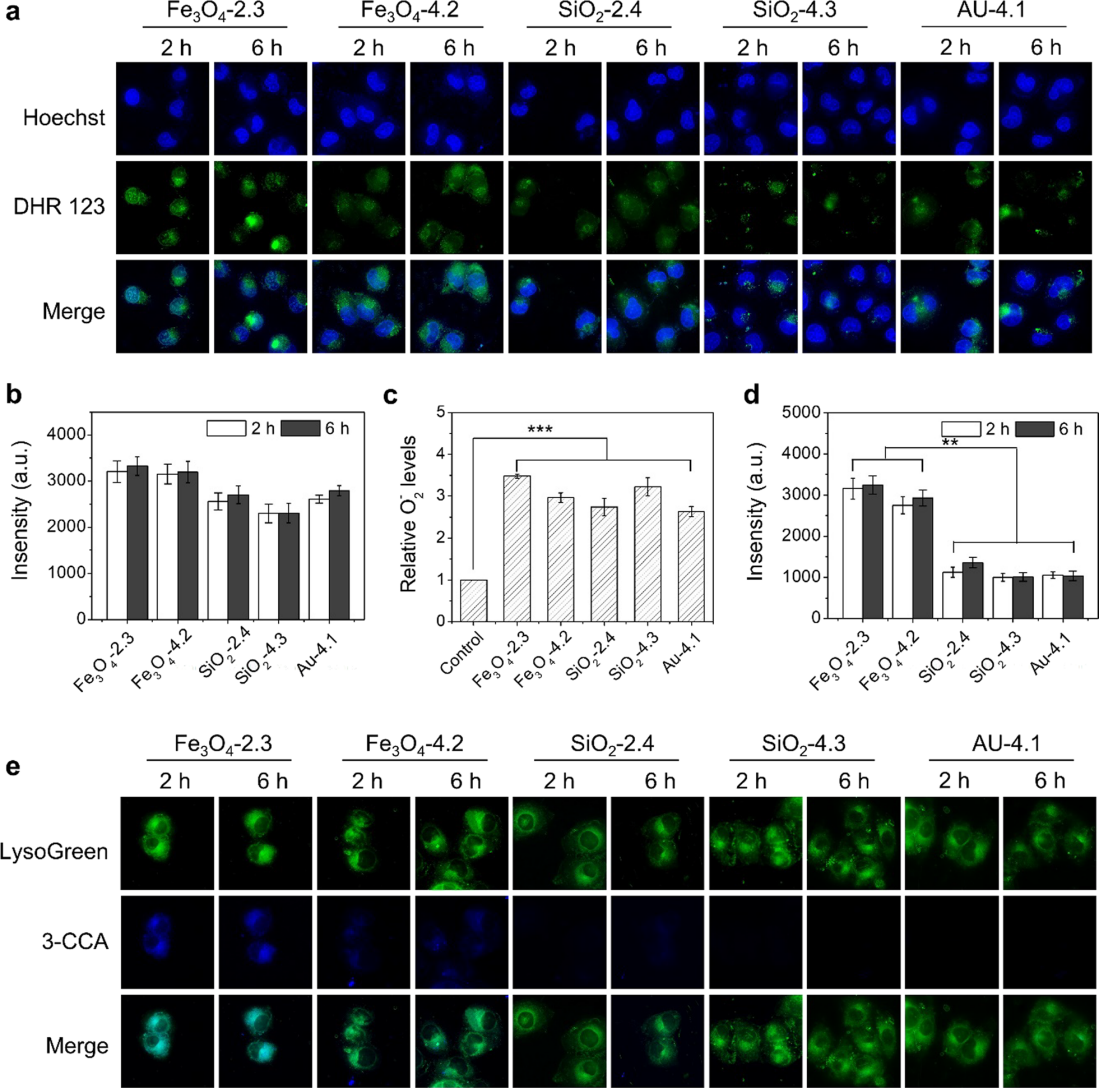
Identification of the generated ROS. **a** Fluorescence images of MCF-7 cells treated with different-sized Fe_3_O_4_, SiO_2_, and Au NPs and stained with DHR123 to characterize the generated H_2_O_2_. Green fluorescence: DHR123, an indicator of H_2_O_2_; blue fluorescence: Hoechst 33342. **b** The corresponding fluorescence intensities of DHR123 in MCF-7 cells after incubation with different NPs for 2 h and 6 h. **c** Generated O_2_^−^ in cells determined by formazan formation using a WST-1 assay kit after incubation with NPs for 24 h. **d** Fluorescence intensities and **e** fluorescence images showing the generated ·OH in the cells using 3-CCA as probe. Green fluorescence: LysoGreen; blue fluorescence: 3-CCA, an indicator of ·OH. All of the data were presented as the mean ± SD (n = 3). *P* values: **P < 0.01, ***P < 0.001

We further investigated the type of ROS induced by the different kinds of nanoparticles. We firstly detected H_2_O_2_ using dihydrorhodamine 123 (DHR123) as indicator [29]. As shown in Fig. 4a, fluorescence signal could be seen in all the groups, indicating H_2_O_2_ could be induced by Fe_3_O_4_, SiO_2_, and Au NPs. We also noticed that the fluorescence intensity of Fe_3_O_4_ NPs was slightly stronger than the other two groups (Fig. 4b), which might be due to the superior capability in inducing oxidative stress. We then measured the O_2_^−^ in cells using a WST-1 assay, which could be oxidized to form orange formazan [30]. As shown in Fig. 4c, there was significant increase of O_2_^−^ concentration in all NPs treated groups. These results were in consistence with that of H_2_O_2_. Generally, the generated O_2_^−^ could be catalyzed by superoxide dismutase (SOD) to produce H_2_O_2_ [31, 32], resulting in the elevation of H_2_O_2_ concentration. The above results demonstrate that the formation of O_2_^−^ and H_2_O_2_ can be induced by ultrasmall Fe_3_O_4_, SiO_2_, and Au NPs.

The generation of ·OH was considered to be another reason for the high toxicity of the ultrasmall Fe_3_O_4_ NPs. Hence, we investigated the intracellular generation of ·OH in MCF-7 cells. As presented in Fig. 4d, e, blue fluorescence of 7-hydroxycoumarin could be found in Fe_3_O_4_ NPs treated cells and the fluorescence signal in 2.3 nm Fe_3_O_4_ NPs groups was much higher than that of 4.2 nm NPs. The enhanced fluorescence signal in 2.3 nm Fe_3_O_4_ NPs group might be due to quicker release of Fe^2+^ from 2.3 nm Fe_3_O_4_ NPs and the subsequent quicker catalytic reaction. In contrast, no distinct fluorescence could be observed in SiO_2_ and Au NPs groups. Meanwhile, we observed a highly co-localized fluorescence of 7-hydroxycoumarin and Lysotracker, demonstrating that the Fe_3_O_4_ NPs was endocytosed via an endolysosomal pathway (Fig. 4e). Although the Fe_3_O_4_ NPs were found in the nucleus (Additional file 1: Fig. S4), no fluorescence could be detected in the nucleus. The possible reason for this phenomenon might be the acidic environment of the lysosomes, which promoted the dissolution of the Fe_3_O_4_ NPs and accelerated the generation of ·OH.

### In vivo toxicity of the Fe_3_O_4_ NPs

**Fig. 5:**
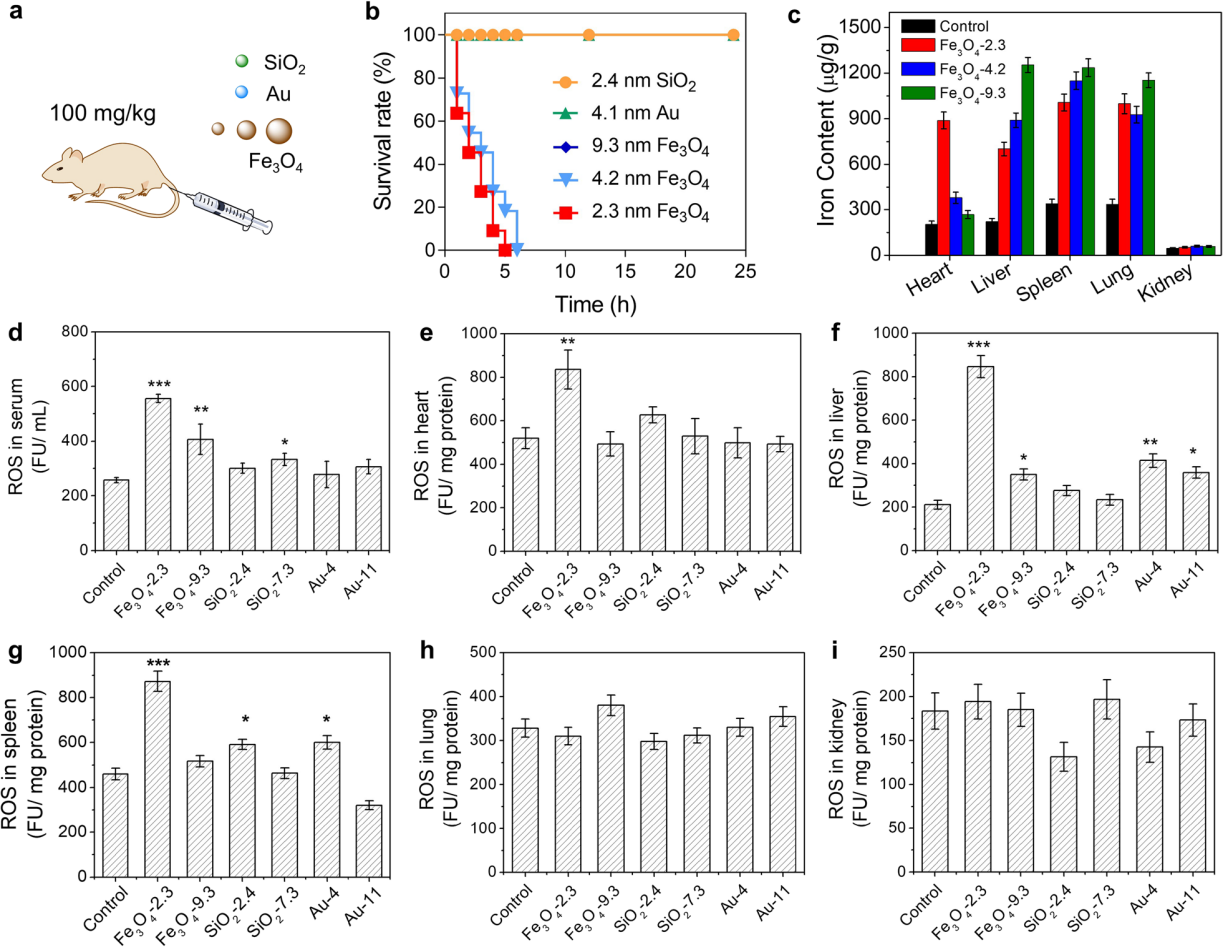
Evaluation of the toxicity in vivo. **a** Schematic illustration of the treatment. The SiO_2_ (2.4 nm) and Au (4.1 nm) and Fe_3_O_4_ (2.3, 4.2, and 9.3 nm) NPs were intravenously injected at a dosage of 100 mg/kg. **b** Survival rates of the mice over a period of 24 h after intravenous injection. **c** Quantitative determination of iron in different organs obtained by ICP-AES showing the biodistribution of Fe_3_O_4_ NPs (n = 3). The concentration of total ROS in serum (**d**), heart (**e**), liver (**f**), spleen (**g**), lung (**h**) and kidney (**i**) after intravenous injection of different-sized Fe_3_O_4_, SiO_2_ and Au NPs (100 mg/kg) for 2 h. The ROS was determined using DCFH as an indicator. All of the data were presented as the mean ± SD (n = 3). *P* values: *P < 0.05, **P < 0.01 and ***P < 0.001 compared with the corresponding control group

The in vivo toxicity of the NPs was studied by intravenous injection of SiO_2_ (2.4 nm) and Au (4.1 nm) and Fe_3_O_4_ (2.3, 4.2, and 9.3 nm) NPs at a dosage of 100 mg/kg (Fig. 5a). The survival rate over 24 h post injection was shown in Fig. 5b. Mice injected with ultrasmall Fe_3_O_4_ (2.3 and 4.2 nm) NPs were all killed. By contrast, no mice were killed in the groups treated with large Fe_3_O_4_ (9.3 nm), SiO_2_ (2.4 nm) and Au (4.1 nm) NPs. After injection of Fe_3_O_4_-2.3 nm NPs, significantly elevated levels of alanine aminotransferase (ALT) and aspartate aminotransferase (AST) were observed, suggesting the hepatic toxicity (Additional file 1: Figs. S7).

To explore the possible in vivo toxic mechanism, we tried to understand the distribution of the NPs when the mice were dead. We elevated the dosage to 500 mg/kg. At such a dosage, the mice will be killed 5 min after the intravenous injection. We could observe the extremely high content of iron element in heart in 2.3 nm Fe_3_O_4_ NPs group, which was 2.35 and 3.33 times to that of 4.2 and 9.3 nm groups, respectively (Fig. 5c). In comparison, the distributions of SiO_2_ and Au NPs in heart were relatively low (Additional file 1: Figs. S8, S9, and S10). Hence, the damage to heart is probably the leading cause of the sudden death. When the injection rate was slow down by injection for 4 times with each interval for 5 min (at total dosage of 100 mg/kg), only one mouse out of five was killed, indicating the decreased toxicity of the Fe_3_O_4_ NPs (Additional file 1: Table S1).

### In vivo generation of ROS

We then evaluated the in vivo ROS levels after intravenous injection of different-sized Fe_3_O_4_, SiO_2_ and Au NPs. The serum and main organs were collected 2 h post injection and the ROS was determined using DCFH-DA as a probe. As shown in Fig. 5d–i, there were remarkable increases of ROS in serum, heart, liver, and spleen in 2.3 nm Fe_3_O_4_ group, which were 2.16, 1.61, 4.03, and 1.9 times as high as that of control. The repeated injection would cause the further increase of the ROS (Additional file 1: Fig. S11). We also notice that the ROS level in 2.3 nm Fe_3_O_4_ group was much higher than that of 9.3 nm Fe_3_O_4_ group. For SiO_2_ and Au NPs, the ROS level of ultrasmall group in liver, heart, and spleen was also higher than larger counterparts. However, the ROS levels in SiO_2_ and Au NPs groups were notably lower than that in Fe_3_O_4_ group. This result might be due to the quick elimination of ROS by ROS-scavenging enzymes, including SOD, catalase, and glutathione peroxidase. These enzymes help the cells to regulate the O_2_^−^ and H_2_O_2_ at a relatively low level and prevent them from being damaged. However, some kinds of ROS, such as ·OH and peroxynitrite (ONOO^–^), cannot be scavenged by any known enzymes in vivo, resulting in high toxicity. Considering the quick scavenge of O_2_^−^ and H_2_O_2_, it is reasonable to deduce that the ROS in 2.3 nm Fe_3_O_4_ group shall be the ·OH.

**Fig. 6:**
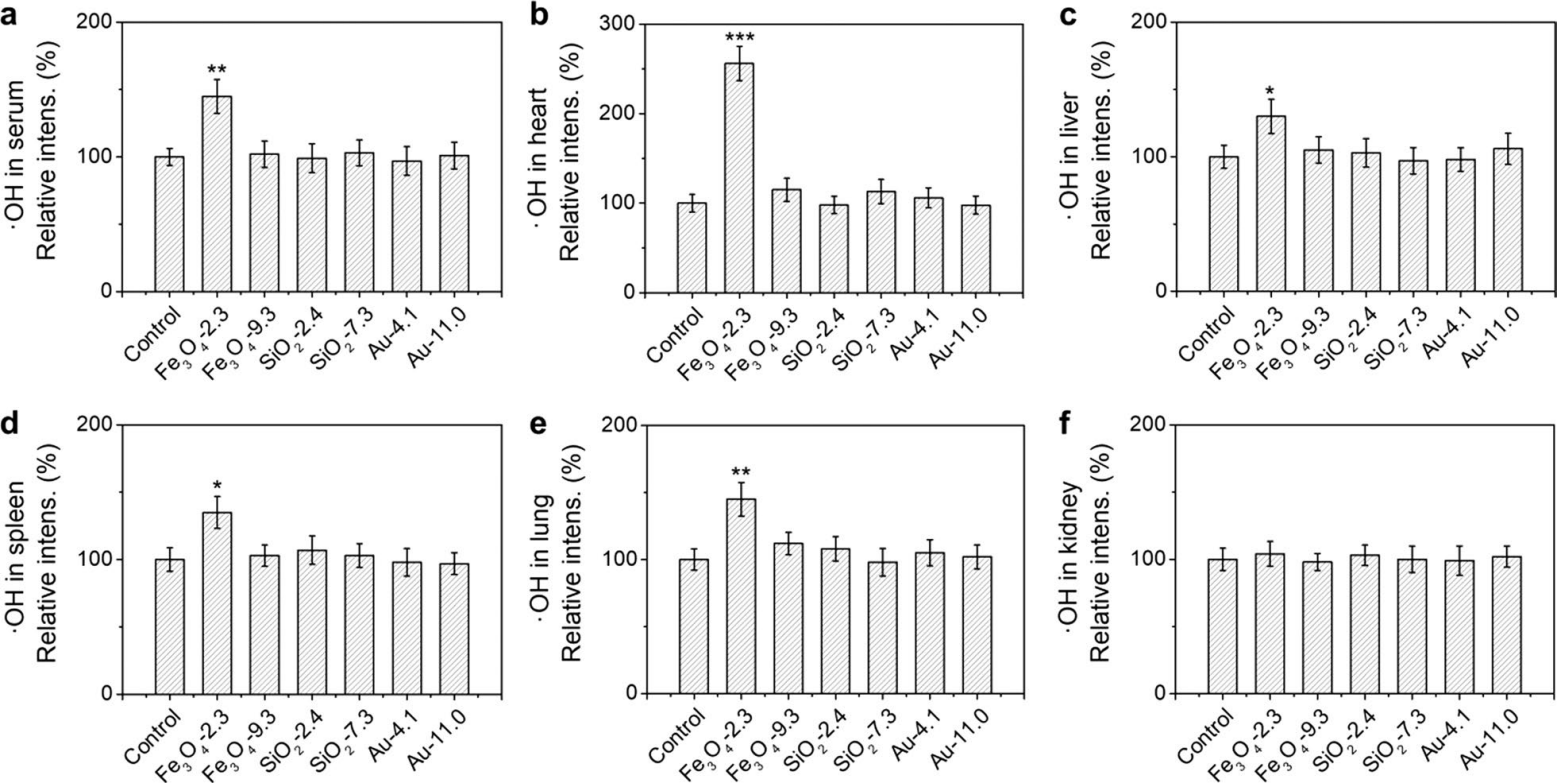
Detection of ·OH in vivo. The concentration of ·OH in serum (**a**), heart (**b**), liver (**c**), spleen (**d**), lung (**e**) and kidney (**f**) after intravenous injection of different-sized Fe_3_O_4_, SiO_2_ and Au NPs (100 mg/kg) for 2 h and 3-CCA for another 1 h. The ROS was determined using 3-CCA as an indicator. All of the data were presented as the mean ± SD (n = 3). *P* values: *P < 0.05, **P < 0.01 and ***P < 0.001 compared with the corresponding control group

To explore the type of ROS in vivo after administration of the NPs, we investigated the concentration of ·OH in serum and main organs using 3-CCA as indicator. After administration of 2.3 nm Fe_3_O_4_ NPs for 2 h, the ·OH concentrations in serum, heart, liver, spleen and lung increased significantly (Fig. 6). In particular, the ·OH concentration in heart increased by 2.56 times. The extremely high ·OH level should be attributed to the high accumulation of 2.3 nm Fe_3_O_4_ NPs in heart and resulting high Fe^2+^ concentration. It has been reported that the heart was sensitive to the ROS, since the oxidative stress can drive heart failure, cardiac arrhythmias, and sudden cardiac death by regulating the ion channels [33, 34]. Hence, the ultrasmall NPs caused death is probably due to the cardiac oxidative stress. We could observe the prominent cardiotoxicity and also the pulmonary toxicity in the H&E stained images as illustrated by the necrotic regions and acute inflammatory cells (Additional file 1: Fig. S12). No noticeable increase of ·OH concentration was observed for the SiO_2_ and Au NPs groups, indicating that the generation of ·OH was induced by the Fe_3_O_4_ NPs. As for 9.3 nm Fe_3_O_4_ NPs, the generated ·OH was negligible because of the low distribution in heart, limited capability in inducing ROS, and the slow release of Fe^2+^.

### Inhibition of the ROS

GSH is an antioxidant that reduces free radicals with the help of glutathione peroxidase 4 (GPX 4). To prove the toxicity was mainly caused by ROS, the mice were pretreated with GSH. As shown in Additional file 1: Fig. S13, the levels of malondialdehyde (MDA) in heart, liver and serum increased by 1.01, 3.41 and 0.63 times, respectively, after the exposure of 2.3 nm Fe_3_O_4_ NPs, indicating the serious lipid peroxidation. Upon the pretreatment of GSH, the malondialdehyde (MDA) levels in heart, liver and serum decreased by 29.3%, 26.4, and 12.5%, respectively. These results suggest the protective effect of GSH to Fe_3_O_4_ NPs induced lipid peroxidation. The GSH could also decrease the levels of ALT and AST (Additional file 1: Fig. S7) and increase the survival rate of the Fe_3_O_4_-2.3 NPs treated mice (Additional file 1: Table S1). These results imply that the toxicity of 2.3 nm Fe_3_O_4_ NPs is caused by the oxidative stress and can be attenuated by antioxidant.

## Discussions

### Generation of the ROS

Ulrtasmall NPs with size < 5 nm, including Fe_3_O_4_, SiO_2_ and Au NPs, can stimulate the cells to generate ROS, while NPs with size over 5 nm do not show remarkable ROS induction effect. Hence, the generation of ROS is dependent on the size of the NPs. It is reported that ultrasmall Au NPs (4.5 nm) can directly penetrate into cell cytoplasm to promote robust ROS production and to activate the NLRP3 inflammasome for Caspase-1 maturation and interleukin-1β production, while only low level of ROS is induced by large nanoparticles [35]. Ultrasmall (< 10 nm in diameter) silica NPs can induce the generation of ROS, and the ROS level increases significantly several hours before cell death [36]. The mitochondrial respiratory chain is a potential source of ROS, and reduced mitochondria membrane induced by the ultrasmall NPs potential leads to increased generation of ROS [37]. As such, various ultrasmall NPs can stimulate the cells to generate the ROS.

### The toxicity of the ultrasmall Fe_3_O_4_

**Fig. 7:**
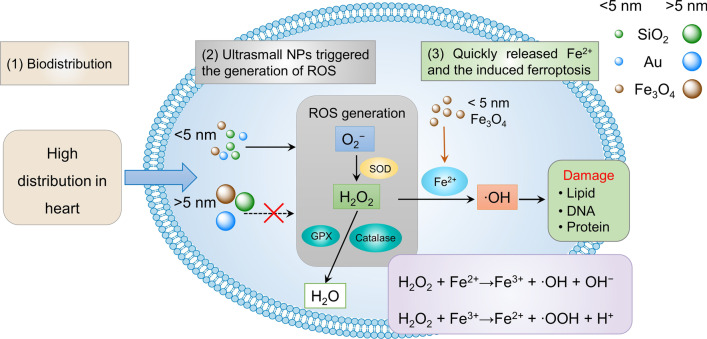
Possible causes of toxicity of USPIONs (< 5 nm): (1) high biodistribution of the ultrasmall NPs in heart; (2) ultrasmall NPs (< 5 nm) trigger the generation of ROS; (3) quick release of Fe^2+^ from ultrasmall NPs induces the ferroptosis based on hydroxyl radicals (·OH)

The toxicity of the ultrasmall Fe_3_O_4_ is believed to be due to the ferroptosis, a type of programmed cell death accompanied by a large amount of iron accumulation and characterized by the accumulation of lipid peroxides. In both in vitro and in vivo experiment, the ·OH is detected in Fe_3_O_4_ group instead of SiO_2_ and gold groups. ·OH is highly reactive and toxic, which cannot be decomposed by any known intrinsic enzymes. By contrast, the O_2_^−^ and H_2_O_2_ can be converted by superoxide dismutase (SOD) and catalase, respectively, to generate non-toxic substances. Although all the ultrasmall NPs (Fe_3_O_4_, SiO_2_ and Au) induce the generation of ROS (O_2_^−^ and H_2_O_2_). Only the Fe_3_O_4_ NPs catalyze the Fenton reaction to produce ·OH and cause lipid peroxidation due to the release of Fe^2+^ (Fig. 7). The quick release of Fe^2+^ from ultrasmall Fe_3_O_4_ NPs facilitates the catalytic reaction. The toxicity is inhibited by the pretreatment of GSH, indicating the toxicity was mainly caused by ROS. To prove the role of released iron in ultrasmall Fe_3_O_4_ NPs induced ferroptosis, deferoxamine can be used as a chelating agent to remove excess iron.

The toxicity is associated with the injection rate. When the total dose was split into 4 doses with 5 min for each interval, the toxicity is significantly reduced. This result may be because when the Fe_3_O_4_ NPs are injected slowly, the released iron will be bonded by the ferritin to regulate the cellular iron concentration at a reasonable level and avoid ferroptosis. However, when the Fe_3_O_4_ NPs are burst injected, the released iron remarkably increases and overloads, causing serious peroxidation. As such, the iron oxide is administrated at a slow rate in clinic. For example, administer Feraheme as an undiluted intravenous injection delivered at a rate of up to 1 mL/s (30 mg/s).

## Conclusions

We found that the ultrasmall Fe_3_O_4_ with sizes of 2.3 and 4.2 nm was highly toxic, while no obvious toxicity was detected for the larger Fe_3_O_4_ (9.3 nm). To explore the toxicological mechanism, different-sized SiO_2_ and Au NPs we synthesized as control and the toxicity was studied. The in vitro results indicated that intracellular ROS was only induced by Fe_3_O_4_ (2.3 and 4.2 nm), SiO_2_ (2.4 and 4.3 nm), Au (4.1 nm), which have small size. The O_2_^−^ and H_2_O_2_ were detected in these three kinds of NPs. While, the ·OH was only detected in the Fe_3_O_4_ NPs treated cells. The ultrasmall Fe_3_O_4_ (2.3 nm) NPs could increase the in vivo ROS and ·OH levels efficiently due to the special capability in inducing ROS and the subsequent Fenton reaction, resulting in the oxidative stress to multiple organs. Among these, heart exhibited highest ·OH level because of the high cardiac distribution of ultrasmall Fe_3_O_4_ NPs, which might be the principal cause of the mice death, since the cardiac oxidative stress can drive cardiac arrhythmias and sudden cardiac death. This study not only illustrates the potential toxicological mechanism of ultrasmall Fe_3_O_4_ nanoparticles, but also presents a potential application of ultrasmall Fe_3_O_4_ in cancer therapy owing to the high toxicity.

## Materials and methods

### Materials

Tetramethyl orthosilicate, trimethoxy-[3-(2-methoxyethoxy) propyl] silane, and chloroauric acid were purchased from Aladdin (China). Hoechst 33342, 3-[4, 5-dimethylthialzol-2-yl]-2, 5-diphenyltetrazolium bromide (MTT) and LysoGreen were obtained from Thermo Fisher. Dihydrorhodamine 123 (DHR123) and coumarin-3-carboxylic acid (3-CCA) were purchased from MedChemExpress. 2′,7′-dichlorofluorescein diacetate (DCFH-DA) and superoxide assay kit were purchased from Beyotime Institute of Biotechnology (China). All reagents were used without further purification. Deionized water prepared by Milli-Q system (Millipore, USA) was used in all the experiments.

### Cell culture and animals

MCF-7 (Human breast carcinoma cancer cell line) cells were purchased from the cell bank of Chinese Academy of Sciences (Shanghai, China). All the cells were cultured in RPMI-1640 medium (Gibco, USA) supplemented with 10% FBS (Sijiqing Biologic, Zhejiang, China), streptomycin (100 U mL^−1^) and penicillin (100 U mL^−1^) at 37 °C in a humidified atmosphere with 5% CO_2_. Male ICR mice (4–5 weeks, 20 ± 1 g) were provided by Laboratory Animal Center of Jiangsu University and all animals were used in accordance with the protocols approved by Guide for the Care and Use of Laboratory Animals.

### Synthesis of Fe_3_O_4_, SiO_2_, and gold NPs

The 9.3 nm Fe_3_O_4_ nanoparticles were synthesized using a DEG-mediated solvothermal reaction [38]. Briefly, anhydrous FeCl_3_ (0.648 g, 4 mmol) and trisodium citrate dihydrate (0.48 g, 1.6 mmol) were dissolved in DEG (40 mL). Afterwards, the mixture was heated to 80 °C under vigorously stirring to form a clear solution. With the addition of anhydrous NaAc (1.0 g, 12.0 mmol), the mixture was transferred into a Teflon-lined stainless-steel autoclave (100 mL capacity). Then, the autoclave was heated at 230 °C for 6 h. After cooling down to room temperature, the sample was centrifuged and washed with ethanol for several times. The resultant black sample was dispersed in water for further use. The 2.3 nm and 4.2 nm Fe_3_O_4_ nanoparticles was obtained at 200 °C for 6 h and 10 h, respectively.

To synthesize 4.2 nm silica nanoparticles, 0.43 mmol of TMOS was added into 10 mL of 0.002 M ammonia aqueous solution [39]. After stirring at room temperature for 20 h, 0.21 mmol of PEG-silane was added and the mixture was stirred at room temperature for another 12 h. After reaction at 80 °C overnight, the solution was cooled down to room temperature and dialyzed (Pierce, molecular weight cutoff = 10,000 Da) in 2000 mL of DI water. The dialysis media was replaced with fresh water every day for total 6 days. As for 2.4 nm silica nanoparticles, the concentration of TMOS was reduced to 0.011 mmol. To prepare 7.3 nm nanoparticles, 0.43 mmol of TMOS was added into 10 mL of 0.002 M ammonia aqueous solution, then the mixture was stirred at 80 °C instead of room temperature for 20 h, while other conditions were identically same as that of 4.2 nm.

Gold nanoparticles (Au NPs) were prepared according to the method developed by Frens [40]. Briefly, 4 mL of 12.7 mM chloroauric acid was added to 49 mL of boiling water, followed by the addition of trisodium citrate solution (5 mL, 38.8 mM). 15 s later, the solution turned to be red and the reaction was quenched in ice bath to obtain 11 nm Au NPs. 4.1 nm Au NPs were prepared by borohydride reduction of the gold salt. Briefly, 1.968 mL of 12.7 mM chloroauric acid was added to 96.432 mL of H_2_O. The solution was kept in ice bath for 10 min. Then, 1 mL of 50 mM trisodium citrate solution was added. 1 min later, 0.6 mL of 0.5 M iced sodium borohydride solution was added to reduce the chloroauric acid to generate 4.1 nm Au NPs. To synthesize 7.2 nm Au NPs, 20 mL of 5 mM hydrogen tetrachloroaurate (III) trihydrate was added into a 3-neck flask and diluted to 90 mL with water. After adding β-cyclodextrin at final concentration of 25 mM, the solution was heated to boiling followed by the rapid addition of sodium citrate (40 mM, 10 mL). 10–40 s later, the solution changed to a murky gray color, suggesting the nucleation of the gold. Within 2 min, Au NPs were formed as indicated by the color of burgundy red. All Au NPs were washed with H_2_O and concentrated by centrifugation. To observe the morphology and structure, the NPs stock solutions were diluted and dropped on TEM grid. After air-drying, the samples were characterized by JEOL transmission electron microscope (JEM-2100) at an acceleration voltage of 200 kV. The size distributions of the nanoparticles were calculated by measuring 150 particles randomly selected from the TEM images.

### Particle aggregation in culture medium

@@@The aggregation of the nanoparticles was determined in the cell-free growth medium at 37 °C for 2 h. Different sized NPs were separated by ultrafiltration centrifuge tube (< 10 kDa, with pore of 2.9 nm) and 10 nm filter membrane (GVS polycarbonate membrane). Total Fe/Si/Au analysis of dispersions and their particle-sizing fractions was conducted by inductively coupled plasma optical emission spectroscopy (ICP-AES). The element analysis thus revealed the percentages in the dispersion below 2.9 nm, between 2.9 and 10 nm, and above 10 nm (considered aggregation) in size.

### Release of Fe^2+^ from Fe_3_O_4_ NPs and the Fenton’s reaction

The release of Fe^2+^ from Fe_3_O_4_ NPs were determined by dispersing Fe_3_O_4_ NPs in PBS with various pH values of 4.0, 5.0, 6.0, and 7.4. 2 h later, the mixtures were centrifugated at 12,000 rpm for 10 min and the supernatants were titrated with 1 M potassium permanganate solution to measure the amount of released Fe^2+^.

To measure the catalysis of Fe_3_O_4_ NPs for the H_2_O_2_, 100 μL of H_2_O_2_ (1 M) and 2 mg Fe_3_O_4_ NPs were dispersed in 2 mL of PBS (pH 5.0). After reaction for 2 h, 1 mL of 3-CCA (0.01 M) was added. 30 min later, the fluorescence (Ex: 350 nm, Em: 450 nm) of the solution was determined using fluorospectro-photometer (F4000, Japan).

### Cellular uptake of the NPs

Three kinds of NPs with different sizes were incubated with MCF-7 cells for 2 h at a concentration of 50 μg/mL. Then, the cells were collected with the help of trypsin and homogenized. After digestion with aqua regia, the concentrations of Fe/Si/Au were measured by ICP-AES.

The uptake of the NPs was also characterized by laser scanning confocal microscopy (LSCM). The NPs were labeled with doxorubicin (DOX), a fluorescent drug that can enter into the nucleus. DOX labeled NPs were prepared by mixing 1 mL of DOX (1 mg/mL) with 2 mL of NPs (2 mg/mL) overnight. Unbounded DOX was removed by centrifugation and washed with water for three times. Then, the DOX labeled NPs were cultured with MCF-7 cells at DOX concentration of 10 μg/mL. After incubation for 0.5 h or 1 h, the culture media was removed and the cells were washed with PBS for three times. The cells were stained with LysoGreen (1 μM)and Hoechst 33342 (10 µg/mL) and observed with LSCM.

### Cytotoxicity of the Fe_3_O_4_ NPs

Cytotoxicity of the Fe_3_O_4_ NPs was assessed using 3-[4,5-dimethylthialzol-2-yl]-2,5-diphenyltetrazolium bromide (MTT) method. MCF-7 cells were seed into 96-well plate at a density of 5 × 10^3^ cells/well and cultured overnight. Then, Fe_3_O_4_ NPs with various concentrations were added and incubated for another 24 h. After removing the NPs, the cells were washed with PBS and the cell viability was determined by MTT using an ELISA plate reader (Thermo Multiskan Spectrum, USA). For GSH pretreatment groups, the cells were incubated with GSH for 4 h at a concentration of 300 μg/mL. Then, the culture media was removed and the cells were treated with H_2_O_2_ and Fe_3_O_4_ NPs.

### Determination ROS in MCF-7 cells

The intracellular ROS levels were determined using 2′,7′-dichlorofluorescein diacetate (DCFH-DA) as indicator, which was non-fluorescent and could be oxidized to generate fluorescence. MCF-7 cells (5 × 10^4^) were seeded in single-well plates with glass bottom. After 24 h incubation, the culture medium was replaced with 1 mL of culture medium containing NPs at a concentration of 500 μg/mL and the cells were incubated for another 2 h and 6 h. Then, the medium was removed and the cells were rinsed with PBS for three times. After staining with DCFH-DA (10 μM, 30 min) and Hoechst 33,342 (10 μg/mL, 15 min), the cells were fixed with 4% paraformaldehyde (30 min) and imaged using a laser confocal microscope (Delta Vision TM Elite, GE).

### The in vivo toxicity of NPs

To evaluate the toxicity, the male ICR mice (4–5 weeks, 20 ± 2 g) were randomly divided the into five groups (with 5 mice for each group, n = 5) to receive SiO_2_ (2.4 nm), Au (4.1 nm) and Fe_3_O_4_ (2.3, 4.2, and 9.3 nm) NPs. The NPs were intravenously injected at a dosage of 100 mg/kg. The number of mice surviving over a period of 24 h was recorded. The toxicity of NPs is usually related with the injection rate. We also investigated the toxicity of Fe_3_O_4_ 2.3 nm by intravenously injecting for 4 times with total dosage of 100 mg/kg. The interval for each injection was 5 min. For GSH pretreatment group, the GSH was orally administrated at a dosage of 800 mg/kg. 2 h later, the Fe_3_O_4_ NPs were injected.

To study the damage of the Fe_3_O_4_ NPs to the organs, the 2.3, 4.2, and 9.3 nm Fe_3_O_4_ NPs were injected for 7 continuous days with the dosage of 80 mg/kg for the 1st day and 40 mg/kg for 2ed-7th day. At the 8th and 14th day, mice were sacrificed and the organs were harvested, sectioned, and stained with H&E for histological analyses.

### Serum biochemical analysis

After intravenous injection of Fe_3_O_4_ NPs (100 mg/kg) for 6 h, the mice were anesthetized and the whole blood was sampled from mice eyes. 30 min later, the samples were centrifuged at 3000 rpm for 15 min and the serum was obtained. The enzymes, including ALT and AST ALP were determined using clinical chemistry analyzers (Beckman Coulter, AU5800). For GSH pretreatment group, the GSH was orally administrated at a dosage of 800 mg/kg. 2 h later, the Fe_3_O_4_ NPs were injected.

### Lipid peroxidation assay

After intravenous injection of Fe_3_O_4_ NPs (100 mg/kg) for 6 h, the mice were sacrificed and the serum, heart, and liver were collected. The harvested heart and liver were washed with cold saline and homogenized. The serum and homogenates were determined using MDA assay kits (Nanjing Jiancheng Bioengineering Institute). For GSH pretreatment group, the GSH was orally administrated at a dosage of 800 mg/kg. 2 h later, the Fe_3_O_4_ NPs were injected.

### In vivo distribution of the NPs

High concentration of ultrasmall Fe_3_O_4_ NPs can kill the mice. We tried to understand the distribution of the NPs when the mice were dead. We elevated the dosage to 500 mg/kg. At such a dosage, the mice will be killed 5 min after the intravenous injection. Then the major organs were harvested and weighed. After homogenization and digestion with aqua regia solution for 7 days, the content of Fe was determined using a ICP atomic emission spectrometry (ICP-AES, Varian Vista MPX). The dosages for SiO_2_ (2.4, 4.2 and 7.3 nm) and Au (4.1, 7.2 and 11.0 nm) NPs were also 500 mg/kg. The contents of Si and Au were all determined 5 min post injection.

### Determination of the types of ROS

The intracellular ·OH and H_2_O_2_ were determined by 3-CCA and DHR 123, respectively, which were non-fluorescent under physiological conditions and could be oxidized to be form fluorescent compounds [28]. After treatment with different NPs at a concentration of 500 μg/mL for 2 h and 6 h, the cells were stained with 3-CCA (10 μM, 30 min)/LysoGreen (10 μg/mL, 30 min) or DHR123 (10 μM, 30 min)/Hoechst 33342 (10 μg/mL, 15 min), respectively. Then, the cells were imaged by laser confocal microscope.

To determine the intracellular superoxide, MCF-7 cells were treated with different NPs at a concentration of 500 μg/mL for 24 h. Then, the medium was replaced with 200 μL of superoxide test solution (WST-1, 2-(4-Iodophenyl)-3-(4-nitrophenyl)-5-(2,4-disulfophenyl)-2H-tetrazolium, monosodium salt) and incubated at 37 °C for 3 min. Afterwards, the absorbance was determined at 450 nm by an ELISA plate reader.

### ROS generation in vivo

Male ICR mice (4–5 weeks, 20 ± 2 g) were randomly divided the into seven groups to receive saline, Fe_3_O_4_-2.3, Fe_3_O_4_-9.3, SiO_2_-2.4, SiO_2_-7.3, Au-4.1 and Au-11.0 NPs, respectively. All the NPs was dispersed in saline and intravenously injected at a dosage of 100 mg/kg. After injection for 2 h, mice that were alive were killed and the main organs were harvested. The collected organs were rinsed with iced PBS (pH 7.4) followed by homogenization in 0.1 M PBS. The homogenates were centrifuged at 14,000 rpm for 20 min to remove cellular debris. The supernatants were collected and diluted 10 times for further analysis. To determine the total ROS, 1 mL of the diluted supernatant was mixed with 1 mL of 0.01 M DCFH. 150 μL of the mixture was placed in 96-well plate and detected by a microplate reader. The results were normalized as ROS produced per mg of protein. To determine the hydroxyl radicals, 3-CCA was intravenously injected to the mice at a dosage of 2 mg/kg at 2 h post the injection of NPs. 1 h later, the mice were killed and the main organs were taken for homogenization and determination. The fluorescence of the supernatant was determined using fluorospectro-photometer (Ex: 350 nm, Em: 450 nm). We also studied the influence of repeated injection of 2.3 nm Fe_3_O_4_ NPs on the in vivo ROS. The 2.3 nm Fe_3_O_4_ NPs were intravenously injected to the mice at a dosage of 50 mg/kg for 3 consecutive days. Then the ROS in serum and organs were determined using the above method.

## Supplementary Information

**Additional file 1.** Supplementary table and figures.

## Data Availability

Datasets associated with this study are available from the corresponding authors.
