# Supplementary material for: Ultrasmall iron oxide nanoparticles cause significant toxicity by specifically inducing acute oxidative stress to multiple organs
Source: Part Fibre Toxicol. 2022 Mar 29;19:24. doi: 10.1186/s12989-022-00465-y (PMC8962100; doi:10.1186/s12989-022-00465-y)
Supplement: Supplementary file 1 — Additional file 1. Supplementary table and figures. [file 12989_2022_465_MOESM1_ESM.docx]

Supporting Information

**Ultrasmall Iron Oxide Nanoparticles Cause Significant Toxicity by Specifically Inducing Acute Oxidative Stress to Multiple Organs**

Lin Wu^1^, Wen Wen^1,2^, Xiaofeng Wang^1,2^, Danhua Huang^1,2^, Jin Cao^2^, Xueyong Qi^2^, and Song Shen^2,*^

^1^ *Affiliated Hospital of Jiangsu University, Zhenjiang 212001, China*

^2^ *School of Pharmaceutical Science, Jiangsu University, Zhenjiang 212013, China*

*E-mail addresses: jsdx.shensong@163.com (S. Shen),*


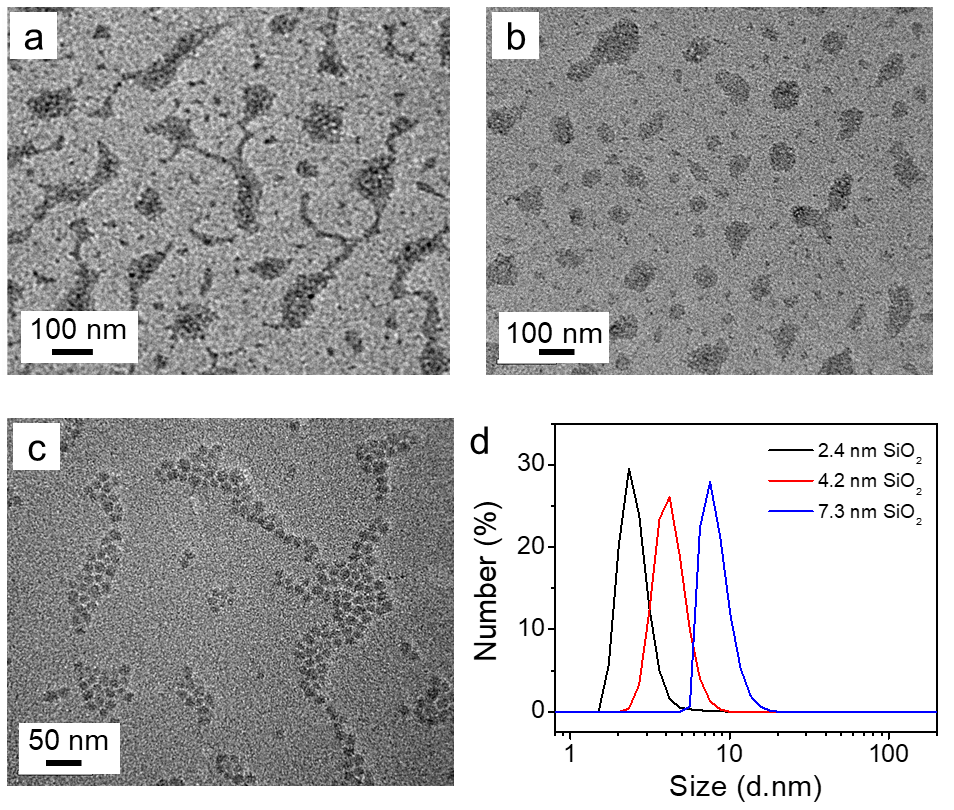


**Figure S1**. TEM images of SiO_2_ NPs with size of a) 2.4 nm, b) 4.2 nm, c) 7.3 nm. d) particle size distribution of the SiO_2_ NPs.


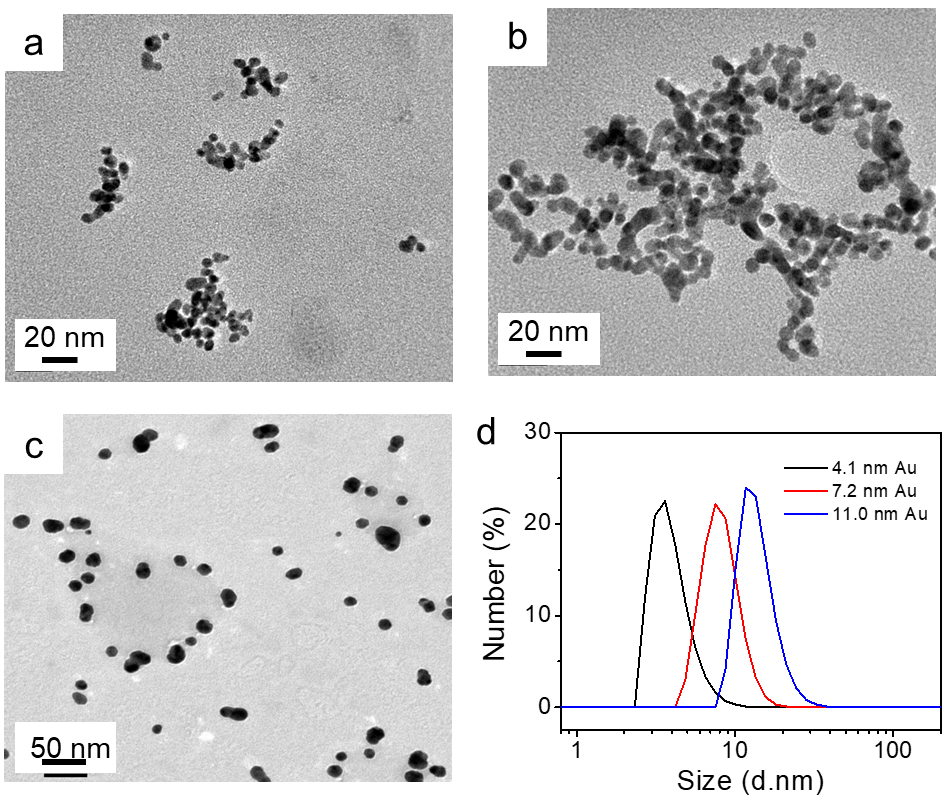


**Figure S2**. TEM images of Au NPs with size of a) 4.1 nm, b) 7.2 nm, c) 11.0 nm. d) particle size distribution of the Au NPs.


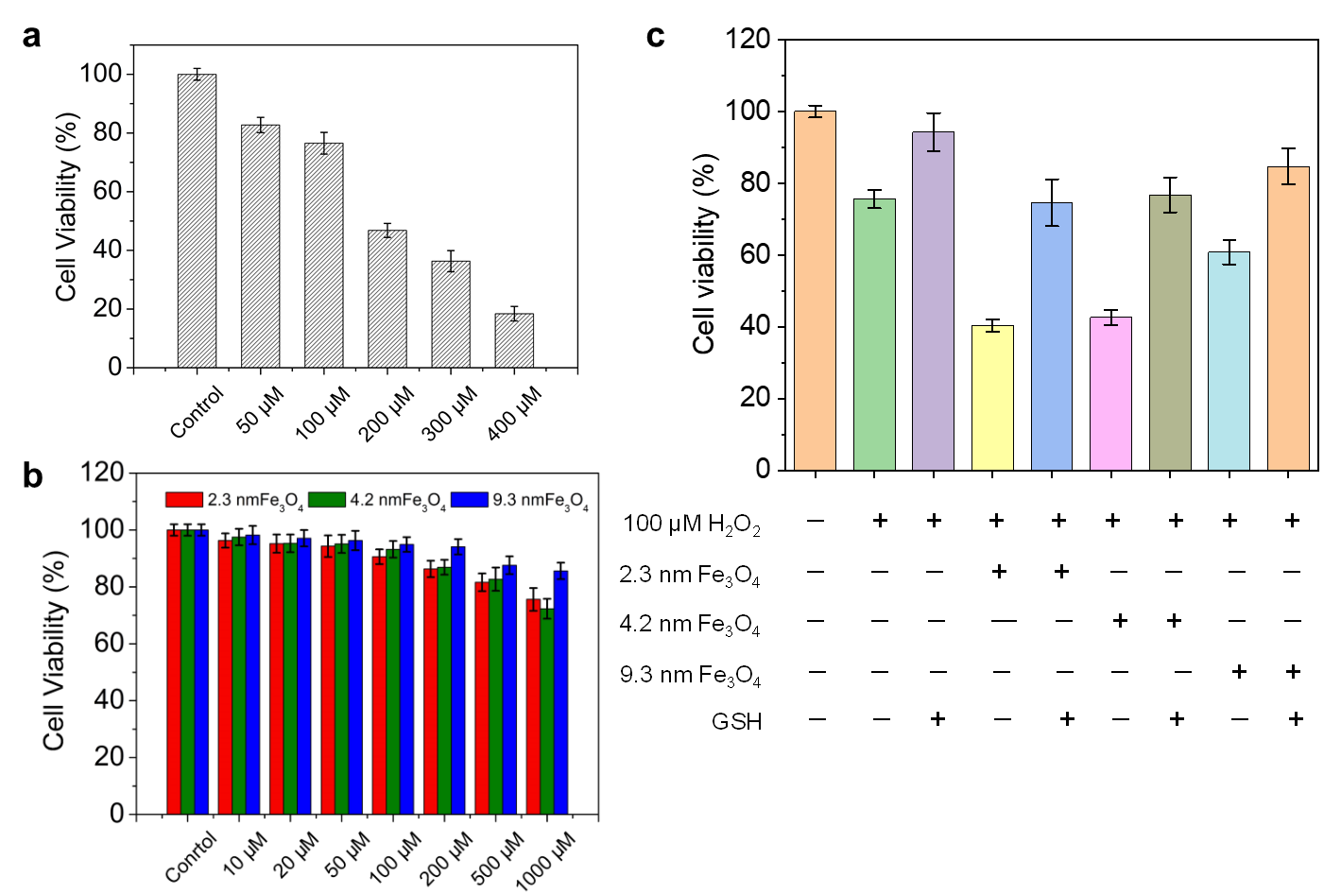


**Figure S3**. a) Toxicity of different concentrations of H_2_O_2_ solution to MCF-7 cells. b) Cell viability of MCF-7 incubated with different-sized Fe_3_O_4_ NPs at different concentrations. c) The cytotoxicity of Fe_3_O_4_ NPs (1000 μg/mL) to MCF-7 cells in the presence and absence of H_2_O_2_ and GSH.


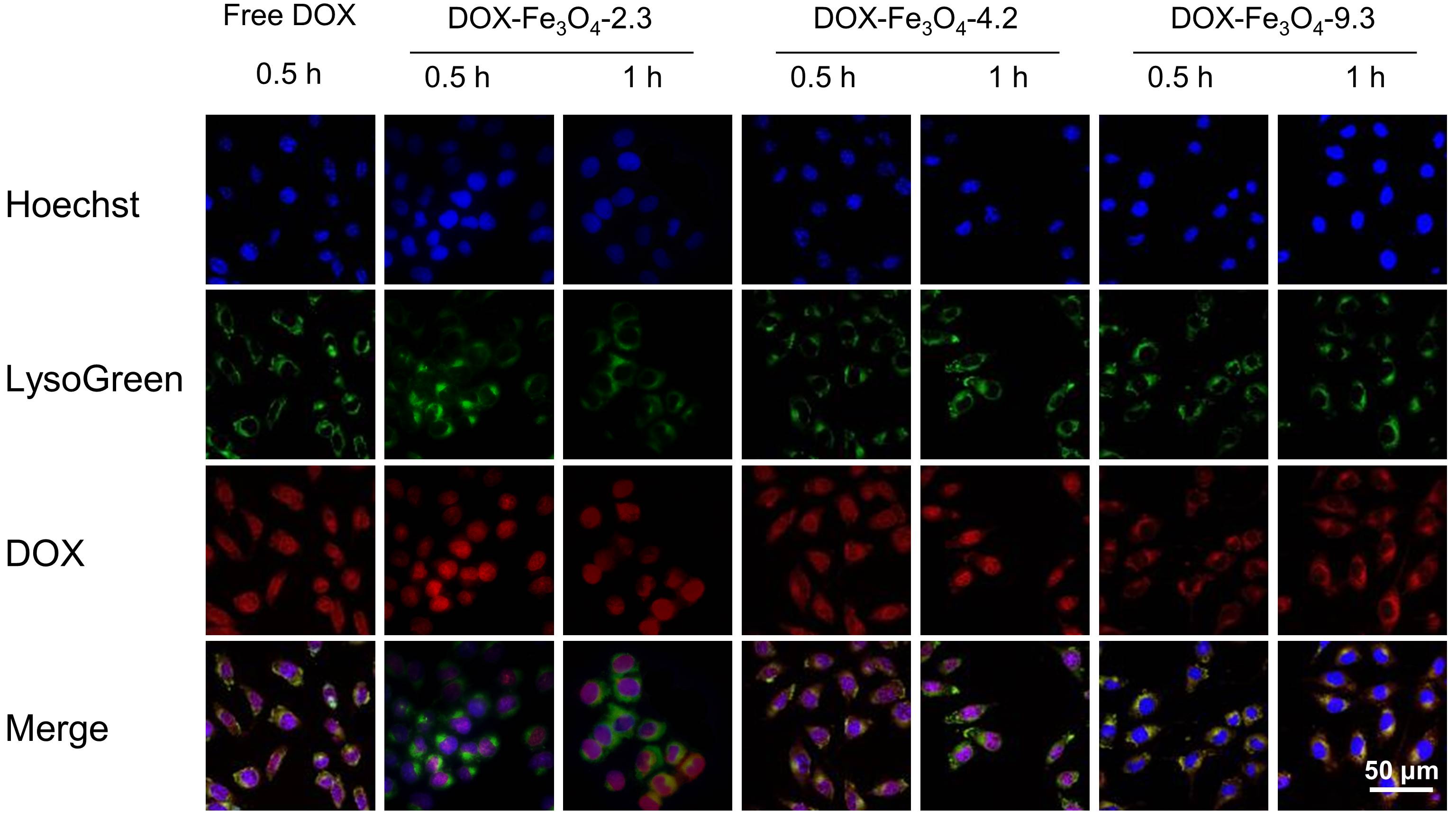


**Figure S4**. Cellular uptake of different size of Fe_3_O_4_ NPs with different incubation time. Red: DOX fluorescence. Blue: Hoechst 33342 staining of nuclei. Green: LysoGreen staining of lysosomes.


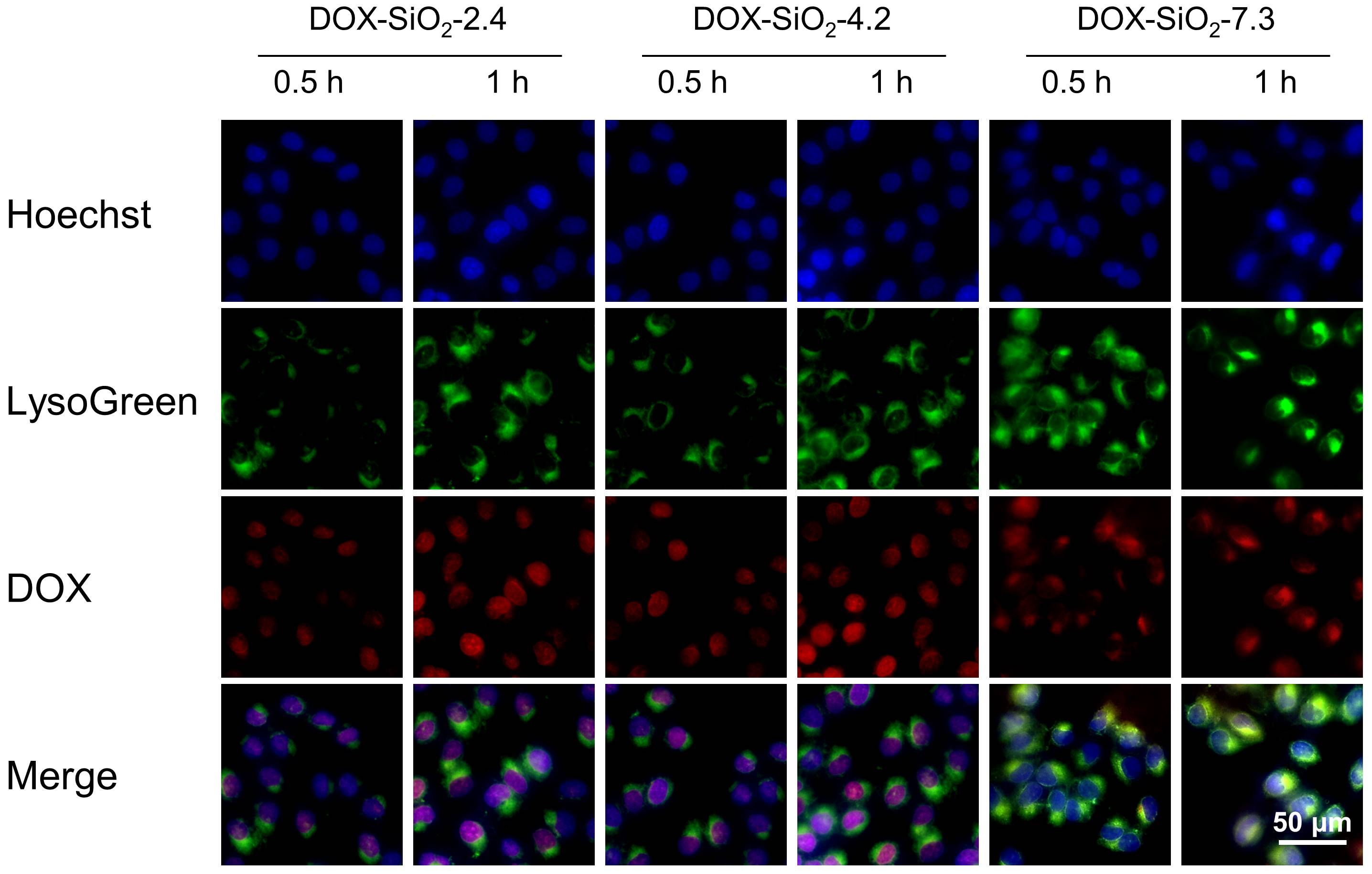


**Figure S5**. Cellular uptake of different size of SiO_2_ NPs with different incubation time. Red: DOX fluorescence. Blue: Hoechst 33342 staining of nuclei. Green: LysoGreen staining of lysosomes.


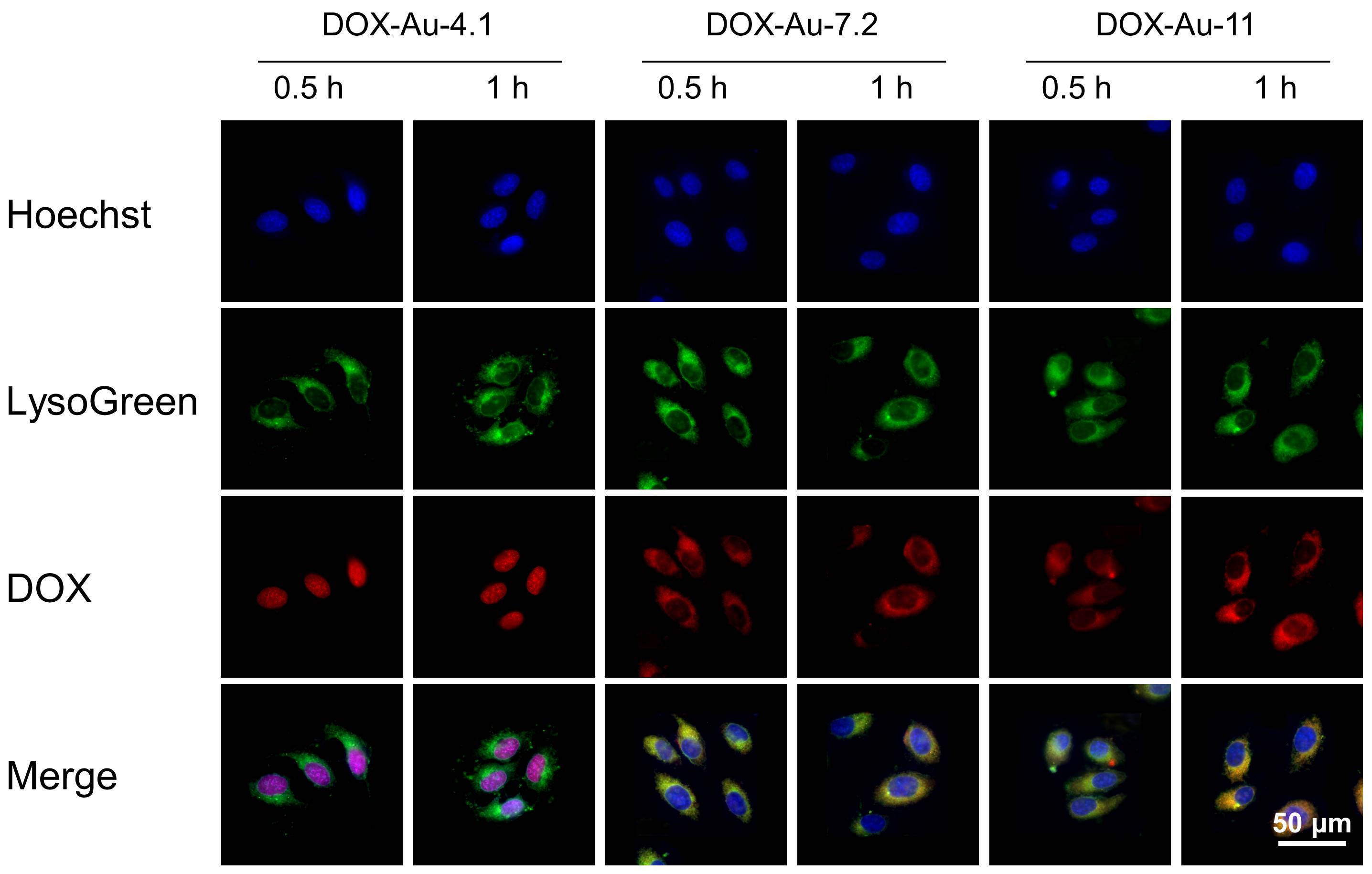


**Figure S6**. Cellular uptake of different sizes of Au NPs with different incubation time. Red: DOX fluorescence. Blue: Hoechst 33342 staining of nuclei. Green: LysoGreen staining of lysosomes.


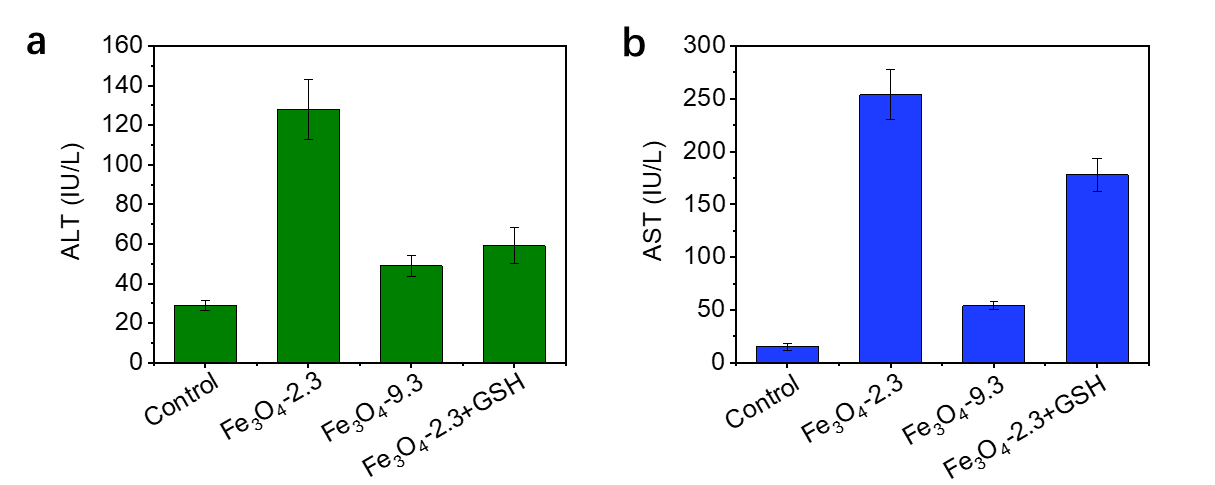


**Figure S7**. The levels of ALT (a) and AST(b) after treatments of Fe_3_O_4_-2.3, Fe_3_O_4_-9.3, and Fe_3_O_4_-2.3+GSH.


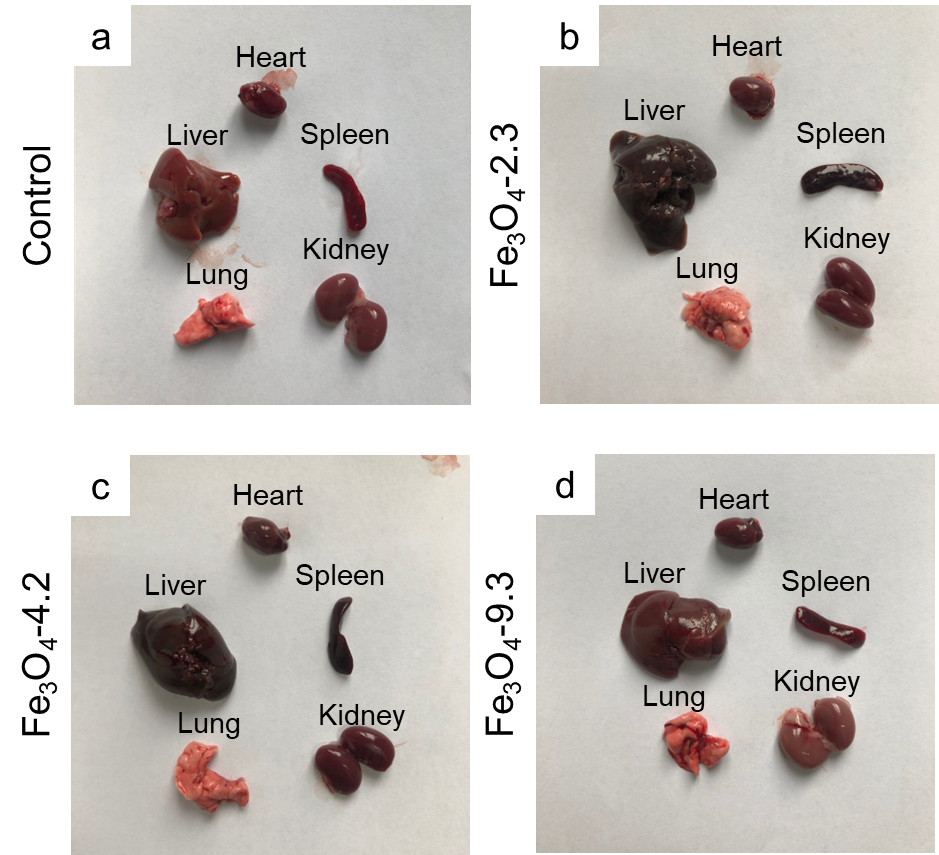


**Figure S8**. Biodistribution of different sizes of Fe_3_O_4_ NPs. Digital images of the major organs collected from mice injected with Fe_3_O_4_ nanoparticles for 24 h.


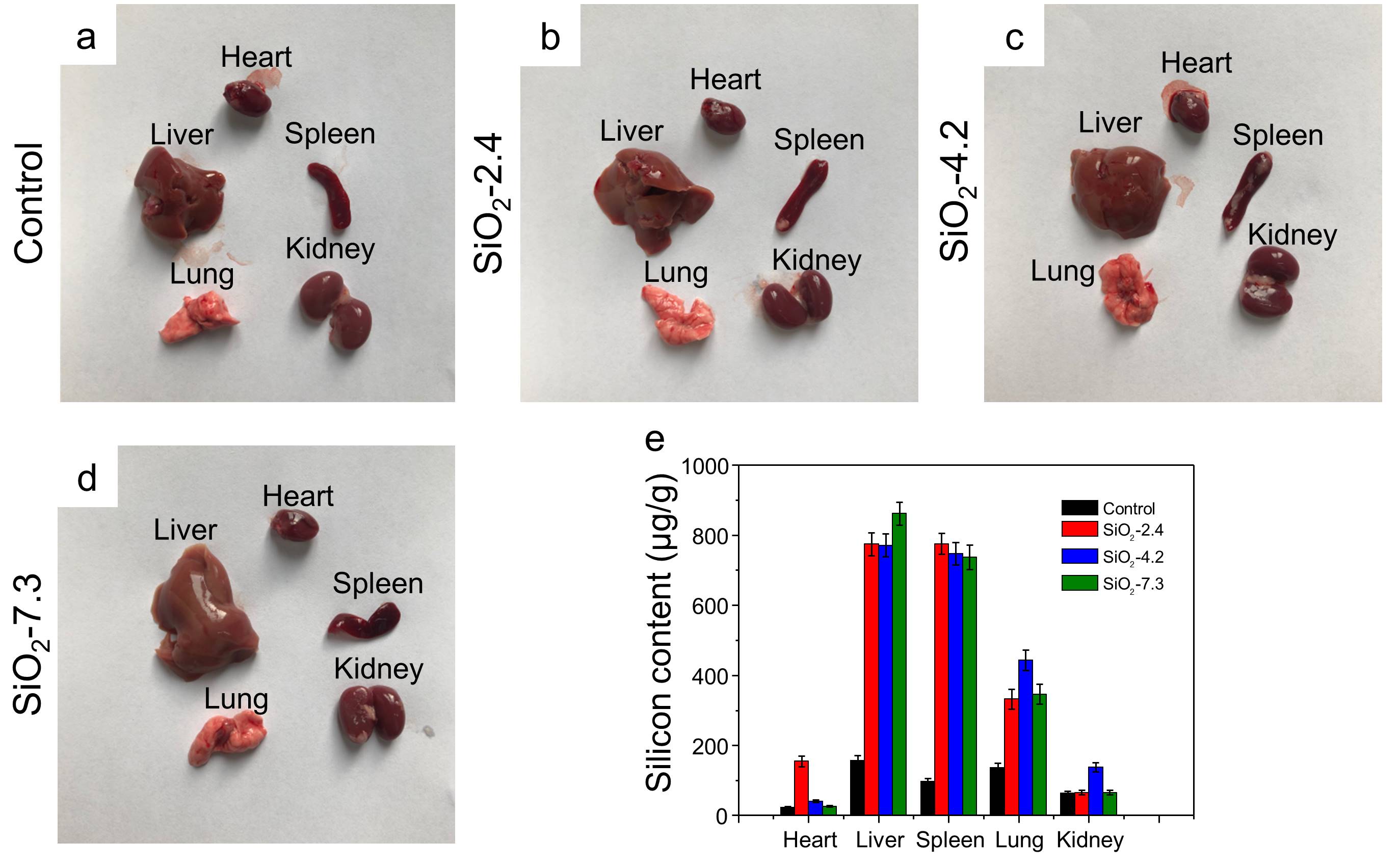


**Figure S9**. Biodistribution of different sizes of SiO_2_ NPs. Digital images of the major organs collected from mice injected with SiO_2_ nanoparticles for 24 h and quantitative determination of silicon in different organs obtained by ICP-AES (n=3).


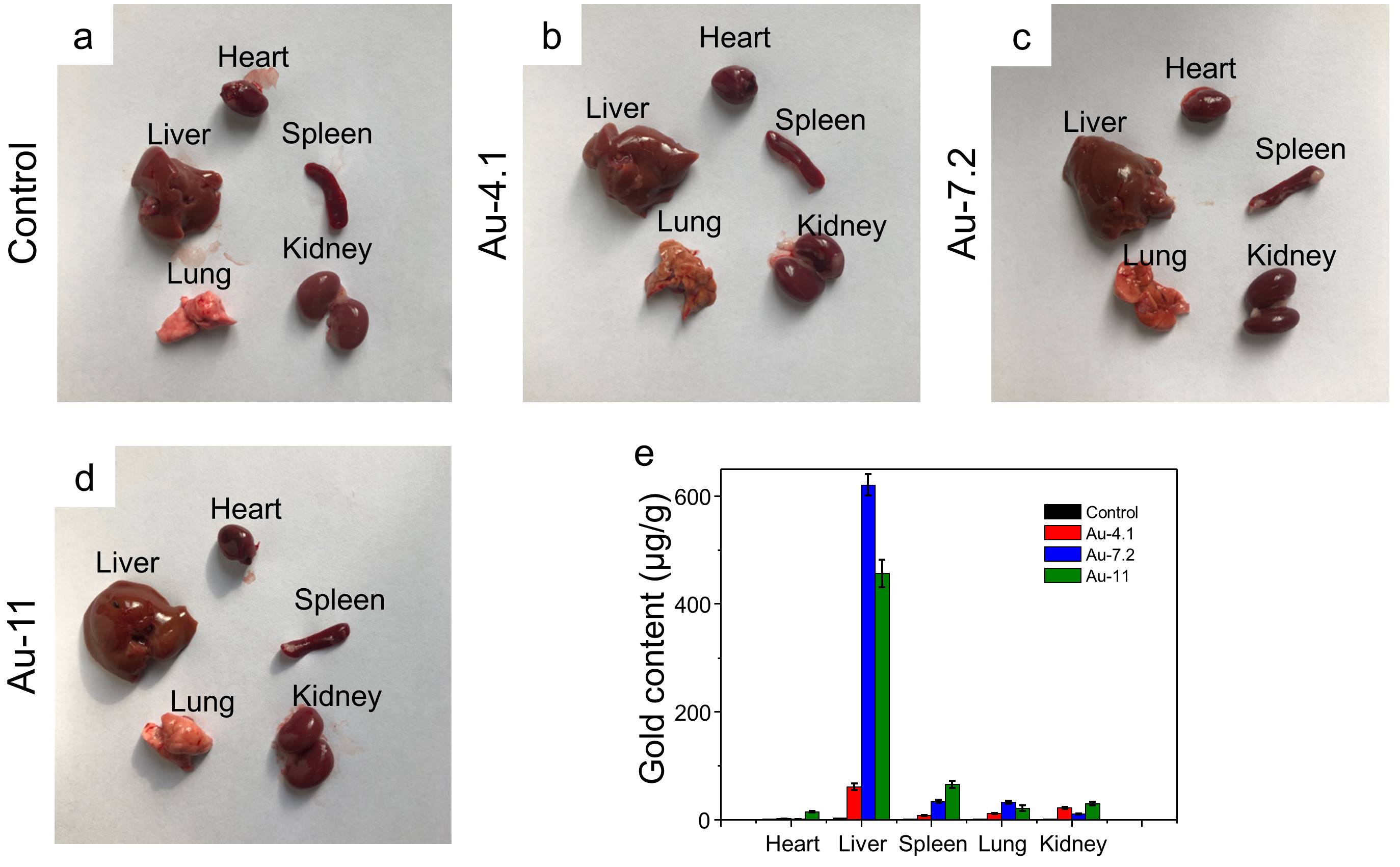


**Figure S10**. Biodistribution of different sizes of Au NPs. Digital images of the major organs collected from mice injected with Au nanoparticles for 24 h and quantitative determination of gold in different organs obtained by ICP-AES (n=3).


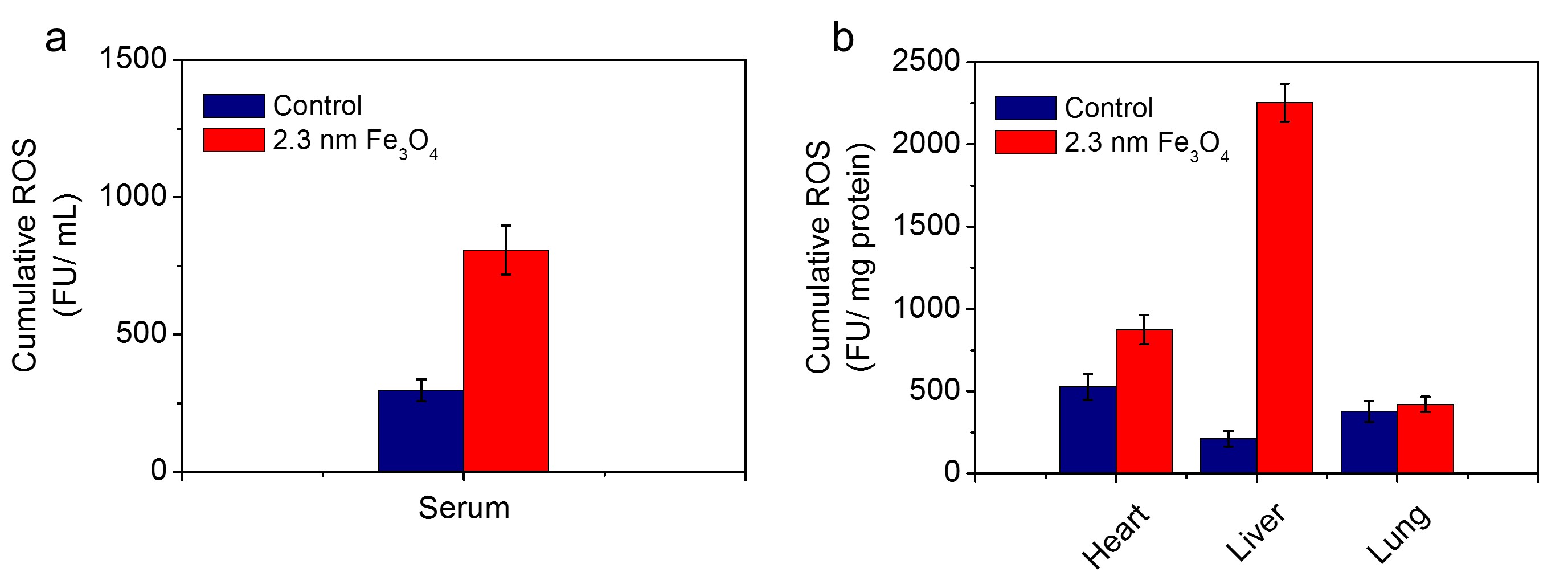


**Figure S11**. ROS in serum (a) and organs (b) after intravenous injection of 2.3 nm Fe_3_O_4_ NPs (50 mg/kg) for 3 consecutive days.


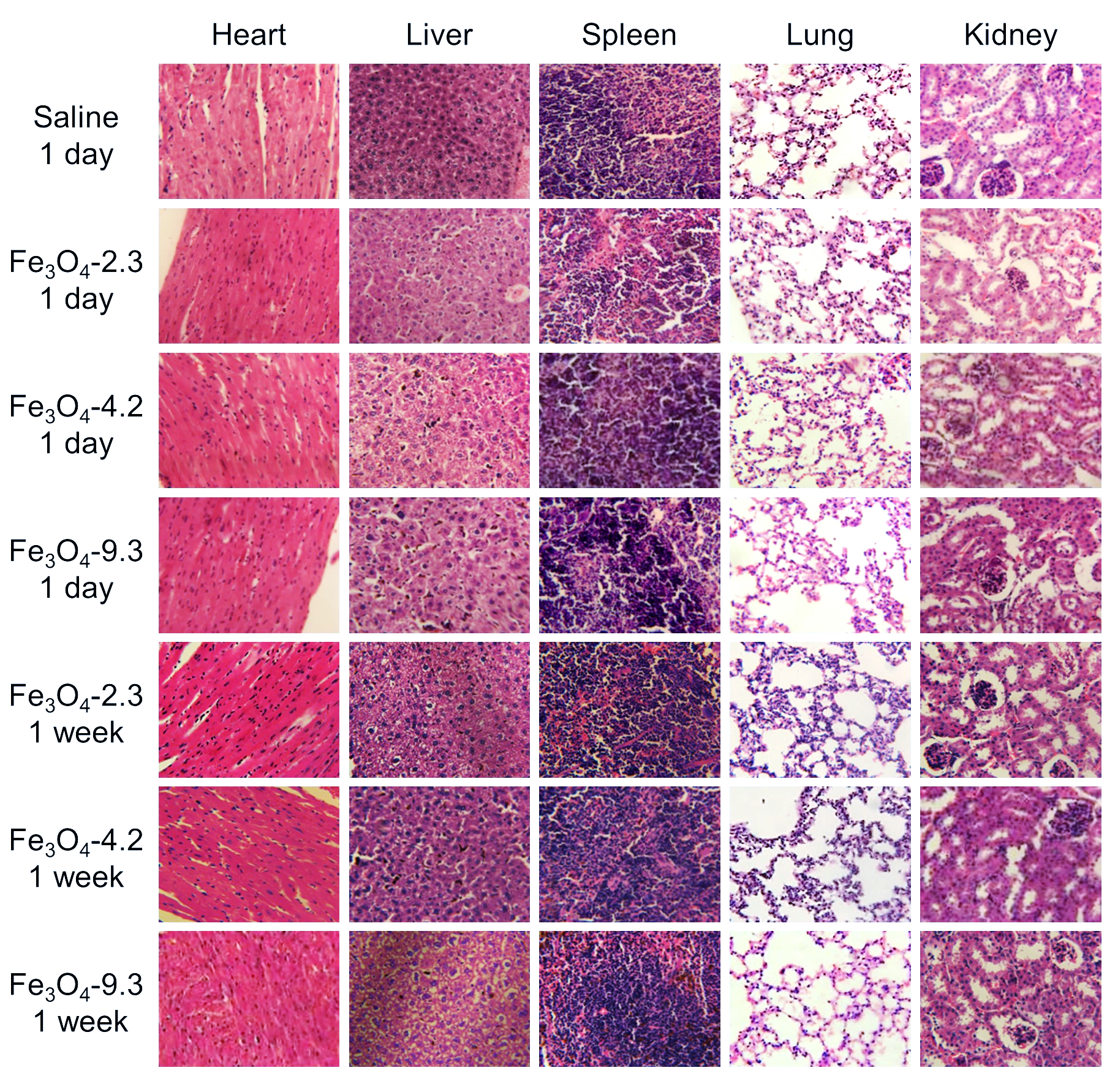


**Figure S12**. H&E stained images of heart, liver, spleen, lung and kidney collected from Fe_3_O_4_-injected and saline-injected mice after injection for 1 day and 1 week.


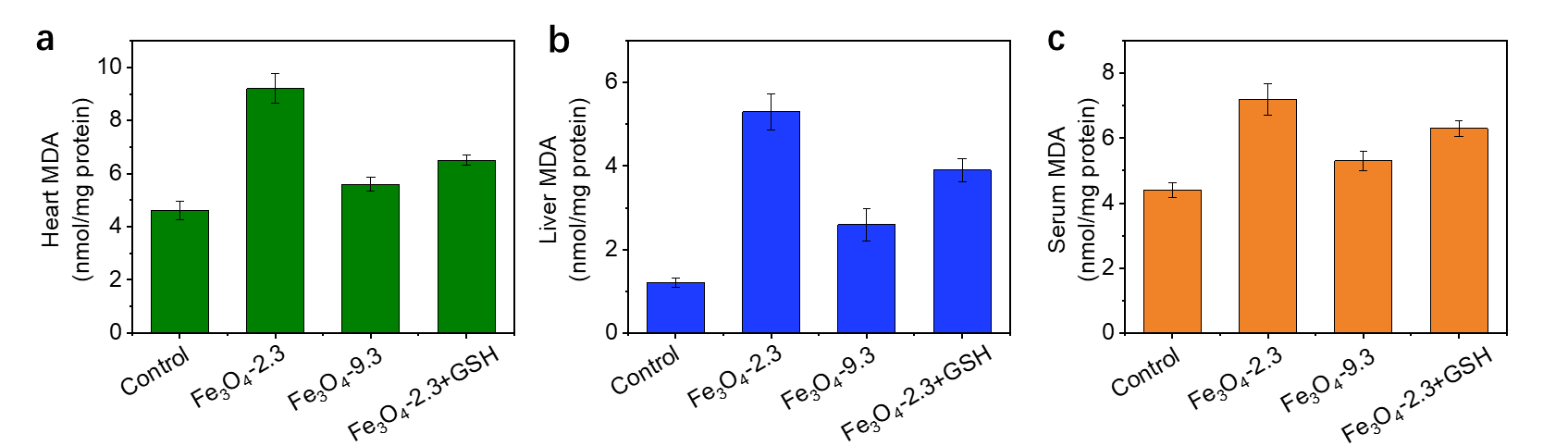


**Figure S13**. The malondialdehyde (MDA) levels in heart (a), liver (b) and serum (c) of the mice after the treatments of Fe_3_O_4_-2.3, Fe_3_O_4_-9.3 NPs and Fe_3_O_4_-2.3+GSH.

Table S1. The surviving rate of mice after injection with Fe_3_O_4_-2.3 or Fe_3_O_4_-2.3+GSH.

|  | Number of dead mice | Number of live mice |
| --- | --- | --- |
| Fe_3_O_4_-2.3, with single injection | 5 | 0 |
| Fe_3_O_4_-2.3, with 4 injections | 1 | 4 |
| Fe_3_O_4_-2.3+GSH | 2 | 3 |
